# Integrating open science education into an undergraduate health professional research program

**DOI:** 10.5195/jmla.2022.1457

**Published:** 2022-10-01

**Authors:** Kevin B. Read, Jessica Lieffers, Merle Massie

**Affiliations:** 1 kevin.read@usask.ca, kevin.read@usask.ca, University Library, Associate Librarian, University of Saskatchewan, Saskatoon, SK, Canada.; 2 jessica.lieffers@usask.ca, jessica.lieffers@usask.ca, College of Pharmacy and Nutrition, Assistant Professor, University of Saskatchewan, Saskatoon, SK, Canada.; 3 merle.massie@usask.ca, Office of the Vice President Research, Coordinator, Undergraduate Research Initiative Research Acceleration and Strategic Initiatives, University of Saskatchewan, Saskatoon, SK, Canada.

**Keywords:** Open science, open science framework, education, curriculum integration, research transparency, open scholarship

## Abstract

**Objective:**

Open science (OS) is a global movement focused on improving research equity, reproducibility, and transparency of research outputs in publicly funded research. While OS education in academia is becoming more common, examples of health sciences librarians providing OS training are not. This paper describes how a librarian collaborated with teaching faculty and a research program coordinator to integrate an OS curriculum into an undergraduate professional practice course and assess students' perceptions of OS after participating.

**Methods:**

A librarian developed an OS-specific curriculum for an undergraduate professional practice course in Nutrition. This course is part of the First Year Research Experience (FYRE) program, which is integrated into 13-week undergraduate courses to introduce students to core elements of the research process in their first year of study by carrying out a research project. The OS curriculum included an Introduction to OS class, a requirement that students share their research outputs in the Open Science Framework, and an assignment asking students to reflect on their experience learning about and practicing OS. Twenty-one of 30 students consented to having their reflection assignment undergo thematic analysis.

**Results:**

Students indicated transparency, accountability, accessibility to research outputs, and increased efficiency as positive attributes of OS. The time commitment, fear of being scooped, and concerns over having research be misinterpreted were considered negative attributes. 90% (n=19) of students indicated that they intend to practice OS in the future.

**Conclusion:**

Based on strong engagement from the students, we believe that this OS curriculum could be adapted to other undergraduate or graduate student contexts where a research project is required.

## INTRODUCTION

Over the last 10 years, open science (OS) has become a global movement improving equitable access to research, reproducibility of research results, and transparency of research methods in publicly funded research. OS is broadly defined as the practice of making all aspects of the research process fully and openly available to increase transparency, improve collaboration, and generate new discoveries through reuse [1]. Calls from research communities to adopt OS practices are increasing: early career researchers have stressed a desire to practice OS because it can result in increased citations, media attention, and new opportunities for collaboration [2, 3]; academic institutions have indicated that OS can accelerate scientific inquiry [4] and have begun adopting OS best practices through policies that support open access, open data, and reproducibility [5]; and researchers across disciplines have called for their fields to adopt OS principles [6–15]. However, while researcher support for OS has grown over time, studies suggest that few actually apply OS in their work due to a lack of knowledge, incentives, and support [16, 17].

To address these gaps, several initiatives have been undertaken in academic settings. For undergraduates, the field of psychophysiology has developed open lab-based training resources focused on using OS in research [18]; microbiology undergraduates in Brazil were provided with OS educational modules in a summer school program [19], and undergraduate research assistants in psychology completed OS readings, practiced preregistering research, and shared data and materials under the supervision of their faculty supervisors [20]. For graduate students and practicing researchers, training has included general OS workshops [21, 22], online courses on reproducible research workflows [23], tailored OS training for PhD students [24], and OS training modules and software support hosted by non-profit organizations [25, 26].

While OS programming at the university level is becoming more common and libraries have been involved in offering OS training [27, 28], examples of health sciences librarians (HSLs) providing OS training are not well reported in the published literature. These gaps have been highlighted in a recent scoping review examining HSL's support for OS [29]. To date, there is evidence of librarians developing training programs focused on specific components of OS such as open data [30], open-source software and community development [31], and reproducible research [32], but there is little evidence of HSL's providing educational interventions that focus on OS as a broad concept in the context of a research project. As the expectation from funders and publishers to adopt OS principles continues to grow, libraries are well positioned to offer training and support to researchers. OS is an extension of scholarly communication and research data management, which makes it a natural addition to library services.

To illustrate one way that librarians can provide comprehensive OS training at academic institutions, this paper will describe how a liaison librarian collaborated with teaching faculty and an undergraduate research program coordinator to integrate an OS curriculum into an undergraduate professional practice course. This project was initiated to advance the University Library's strategic plan to champion open scholarship, to incorporate OS education into a liaison assignment as a proof of concept for other liaison areas, and to promote the library as a leader in OS support across campus.

## METHODS

Seeking pathways for the library to provide OS education and support within an academic institution, a librarian identified an opportunity to integrate OS education into an existing undergraduate Nutrition course. This course is part of a First Year Research Experience (FYRE) program, which is designed to introduce students to core elements of the research process in their first year of study. A description of the FYRE program, its application in the Nutrition course, the OS curricular integration, and our approach to assessing student perspectives of OS are described below.

### The First Year Undergraduate Research Experience (FYRE) Program

The FYRE program is designed to embed a research project requirement into credit-bearing, 13-week undergraduate courses of participating colleges and departments. FYRE projects must follow three components of the research lifecycle: ask a researchable question, address the question using the tools of the discipline, and share results with people beyond the professor. Faculty instructors design the FYRE project according to the needs and expectations of the course.

### FYRE Projects for the Nutrition Professional Practice Course

FYRE was embedded into a first-year professional practice course in Nutrition that trains students to become Registered Dietitians. Students enrolled in this course have on average 1-year (~25%) or 2 or more years (~75%) of university education. The Nutrition FYRE project requires students to work in teams (3-4 students) to conduct a cross-sectional survey investigating the knowledge, attitudes, and/or practices of the university community (students, faculty, and staff). Surveys must address a food, nutrition, and/or dietetic topic of the teams' choice. Teams present their results at the end of the semester in a poster showcase that is marketed to the College of Pharmacy and Nutrition and other courses participating in the FYRE program.

Throughout the semester, students are required to complete the following research checkpoints that follow the research lifecycle:

1. Submit their research question and justification for approval;
2. Draft 3-5 closed-ended survey questions to investigate their research question;
3. Submit their introduction and methods for their poster;
4. Submit progress reports on their data analysis process and poster; and
5. Finalize the poster and prepare the presentation.

More information about the outcomes of the Nutrition FYRE program are described in another publication [33].

### Fall 2020 Semester Open Science Curriculum Integration

In advance of the fall 2020 semester, the librarian submitted a written proposal to the Nutrition course instructor and FYRE program coordinator outlining the integration of an OS curriculum into the course. The proposal included a request to a) teach a 1.5-hour Introduction to OS class, b) incorporate OS-specific checkpoints that coincide with the course's research checkpoints, and c) administer a reflection assignment at the end of the course to gauge students' grasp of OS principles and practices (See Appendix A). The proposal was well received by the program coordinator and course instructor because OS aligns closely with the FYRE program's mission to introduce students to the research process. Another positive attribute of the proposal was that the librarian agreed to take on the majority of the work, ensuring that the instructor and coordinator would not have to drastically alter their existing course content or contribute a significant number of hours to adapting the program.

The following sections describe each component of the OS curriculum integration.

### Introduction to Open Science Class

The class was designed to be a single 1.5-hour session that included didactic lecture, participatory activities, and a class discussion introducing students to the principles and practices of OS. To orient students to OS early in the semester, the class was positioned within the first two weeks of the course and taught virtually using WebEx due to the COVID-19 pandemic. The learning objectives for the class expected students to be able to:

- articulate the difference between closed and open science approaches;
- define open science;
- outline the stages of the research lifecycle.
- identify current open science initiatives; and
- apply best practices in OS.

The lecture portion of the session highlighted differences between open and closed research, examples of OS research in practice [14, 34], how OS can be incorporated into every stage of the research lifecycle, and benefits to using the Open Science Framework (OSF) platform [26] to share outputs throughout the research lifecycle. Activities included students registering for an OSF account, and the class discussion asked students to highlight the differences between open and closed research methods. The class was recorded and uploaded to the learning management system (Canvas) for students to use as a reference tool. The class slides are available in Appendix B.

The OS class was informed by existing material from the FOSTER Open Science Training Handbook [35], the OS knowledge gaps identified in surveys of early career researchers [16, 17], and talking points outlined in editorials from public health calls for OS practices [10, 11]. These foundational materials and concepts were selected because they aligned closely with topics related to public engagement and research transparency discussed in the existing Nutrition course.

### Open Science Checkpoints

**Figure 1:**
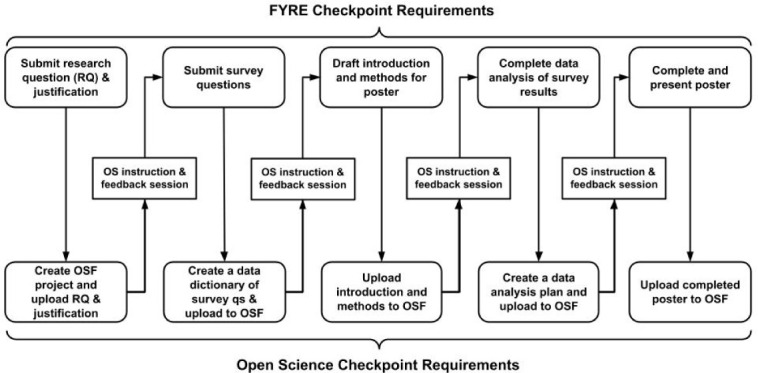
Open science checkpoint activity integration.

After taking the OS class, students were assigned five OS checkpoints to complement the existing FYRE checkpoints (Figure 1). These checkpoints required each FYRE team to:

1. Share their research question and justification at the beginning of their project on the OSF (analogous to submitting a preregistration of their study before initiating it);
2. Use the OSF to share the survey questions they intended to administer and develop a data dictionary to improve the transparency of their raw data;
3. Share their draft introduction and methods section of their poster on OSF;
4. Create and share a data analysis plan on OSF to improve the interpretability of their summarized data and figures; and
5. Upload their final poster while citing their OSF project in the poster itself.

Each OS checkpoint required teams to upload corresponding project documentation to the OSF at the time it was completed in the research process. Projects were also shared using Canvas so that other teams could view and access the research outputs of their classmates as the semester progressed. Completion of each checkpoint was evaluated as pass/fail and was cumulatively worth 1% of students' final grade.

After each checkpoint, the librarian sent structured feedback to each team in Canvas that highlighted areas for improvement. The librarian also attended subsequent classes to provide students with broader feedback on common challenges that emerged from their checkpoints. During these sessions, which were typically 5-10 minutes in length, the librarian also answered questions from the class and introduced the upcoming checkpoint. For checkpoints where a new OS concept or approach was introduced, such as creating a data dictionary or data analysis plan, students were instructed on how to complete these during the session. Students were also given templates (Appendix C and D) to help guide them in developing these research outputs.

At the beginning of the semester, an OS learning module was added to Canvas to provide resources to students as they moved through the checkpoints. This learning module included the OS lecture recording and slides (uploaded after the lecture), links to Panopto videos on getting started with the OSF, data dictionary and analysis templates, and links to resources on file naming conventions and building data dictionaries.

### Open Science Reflection Assignment

After their FYRE project was completed, each student was required to complete an assignment asking them to individually reflect on OS after learning about and practicing it over the course of the semester (Appendix E). Considering this was the first time students were being introduced to OS, we chose a reflection assignment to allow them to internalize these new ideas, draw personal meaning from their experience participating in the checkpoint activities, and express what concepts resonated with them the most [36]. Reflection activities also provide students with the opportunity to reprocess what they learned and make sense of practicing OS in the context of their own beliefs and goals [37]. Our aim was to give students the freedom to speak about OS concepts that mattered to them and learn about their impression of OS when engaging with research for the first time. With this approach in mind, we asked students to respond to the following questions in 250-1,000 words:

- What is your opinion of practicing OS?
- What do you see as the positive aspects of OS?
- What do you see as the negative aspects of OS?
- Do you think you will practice OS in future research projects – why or why not?
- What impact (if any) do you think OS can have on research more broadly?

The assignment was worth 5% of students' final grade and was evaluated based on completeness (responding to each question), breadth and depth of response, organization and clarity, and proper grammar, spelling, and formatting. These assignments were graded by the faculty instructor.

### Analysis of Reflection Assignment

The librarian analyzed the reflection assignments by coding student responses to each question to identify common themes and explore student perceptions of OS. Coding strategies were adapted from methods outlined by Tracy [38] and Saldaňa [39] which focus on thematic analysis and coding categorization. Each reflection assignment was coded separately and manually in Microsoft Word using a conventional content analysis approach [40]. This method was chosen to obtain specific information about OS from the students without preemptively imposing common OS themes that may have influenced their responses. Each time a code was generated, it was defined and reapplied when other student assignments met the definition. Once all reflection assignments were complete, the codes were compiled and tallied in Microsoft Excel. Ethics approval was acquired for this study from the institution's Behavioural Research Ethics Board (ID# 2220), and 21 of 30 students consented to having their reflection assignments used in the analysis. Aggregate data and the codebook are available in Appendix F.

## RESULTS

The goal of this curriculum integration was to introduce students to the principles and practices of OS during their first encounter with the research process. The ability for students to complete the OS checkpoints, combined with the coding results from their reflection assignments provide evidence that they were engaged with the topic and that taking the class had an influence on their intent to adopt OS principles in their future work. This project was also deemed successful with respect to implementing a library-led OS curriculum integration into an undergraduate course, which has been reinforced by an institutional acknowledgement to expand this program to other disciplines. This section will describe the coding outcomes from the reflection assignment.

### Reflection Assignments: Student Perceptions of Open Science

**Figure 2A-D:**
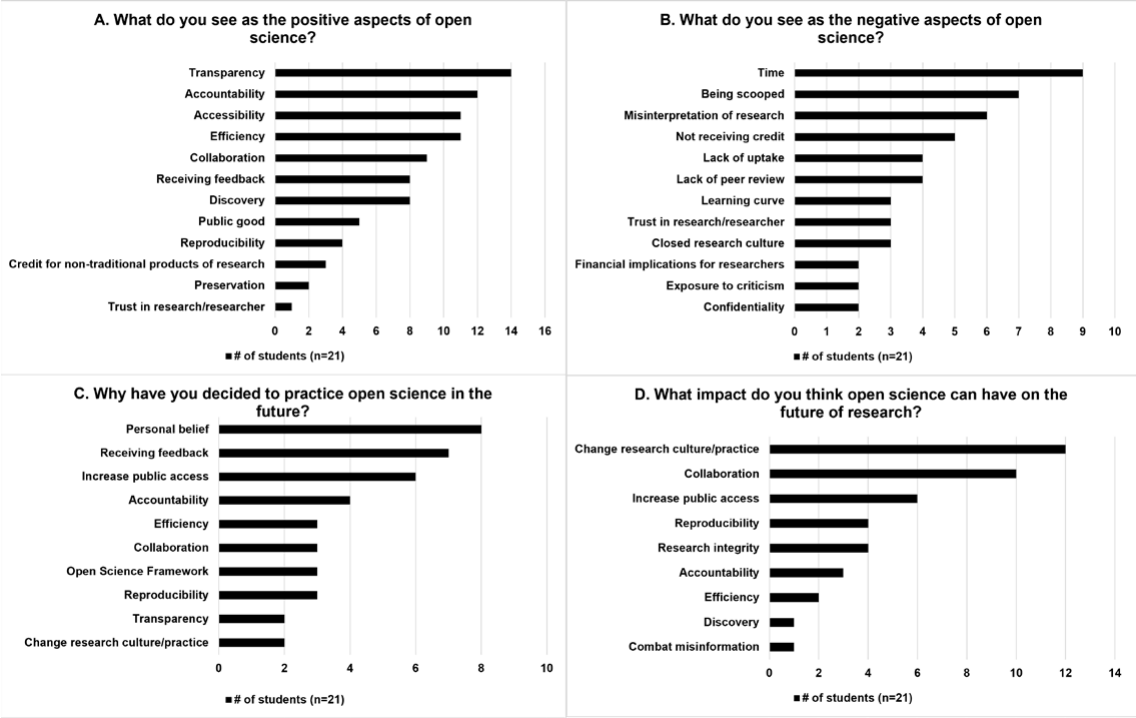
Themed responses on student experiences practicing open science.

In the reflection assignment, students identified transparency, accountability, accessibility to research outputs, and increased efficiency as the most positive attributes of OS (Figure 2A). Quotes taken from the assignment provide examples of how the students engaged with OS concepts. On the topic of transparency, one student provided the following perspective:

**“Throughout every aspect of the project, I [sic] have to ensure that my wording will be understood by someone who is not familiar with research jargon. Not only will this help my research be understood by more people [sic], but it can also decrease the chances of data misinterpretation.”**

With respect to accountability, students commented that knowing their research would be publicly available changed their approach to completing the work:

**“When someone is aware that people can be viewing the process of their research, they maintain a standard of practice that ensures integrity when people view their documents.”**

Students also acknowledged that making their research public placed the onus on them to make sure their outputs were of high quality:

**“Knowing that the work put out will be seen by more people than just the extended community certainly puts pressure to ensure the work is of the highest caliber and standard. I found that open science made for a better overall project because we had to be so specific and professional which made us enormously proud of our final project, and I would wish the same for future science projects that I do.”**

On the opposite end of the spectrum, the amount of time it takes to practice OS, the fear of being scooped, and concerns over having research be misinterpreted were the most common negative attributes expressed by students (Figure 2B). Some students posited that researchers may not be interested in practicing OS because of the time required to do so:

**“Many scientists that do not practice open science likely think of it as a burden on their research as they have to share their progress at each stage which can be time-consuming and potentially require that they put together a few additional documents such as a data dictionary which they may not have needed to do if they were not publicly sharing each stage of the research process.”**

Regarding concerns over being scooped, students took issue with preregistering a study before beginning their project:

**“One aspect of Open Science that I find concerning is the pre-registration process in which a researcher declares their research plan before they have any results. Personally, I would worry that someone else might steal my research idea if it has a unique research focus.”**

Students highlighted concerns about how OS can lead to the misinterpretation of research if only partial research products are made available:

**“…researchers may not upload all the documents that they have. This could lead to misunderstandings of the project for someone who is viewing it from the outside.”**

Similarly, students expressed concerns that not everyone will have the same baseline level of knowledge when engaging with research outputs, which may result in them misunderstanding and even misrepresenting their research:

**“Everybody has different level of understanding. People may not perceive it the way you want them to. So, there is a chance of misleading public by putting it on display for all.”**

In total, 90% (n=19) of students indicated that they intend to practice OS in the future. The reasons provided were that they personally believed it was the right thing to do, hoped to receive feedback earlier on in the research process, and wanted to increase public access to their research (Figure 2C). One student indicated that they would not practice OS in the future because it is not a cultural norm for research at this time. Another cited that they do not plan to pursue a research career and therefore have no intention of adopting OS practices.

Finally, students highlighted that they believe OS will impact the future of research by helping to change the current culture of research, improve collaboration, and increase public access to research outputs (Figure 2D). Several students cited the COVID-19 vaccines as an example of culture change and collaboration:

**“[OS] has the potential to promote sharing of ideas and knowledge in a non-competitive way. It can promote partnership across the globe and foster collaborative approaches to solving problems that face the world daily, for example, the pursuit to find a safe and effective COVID-19 vaccine.”**

Others addressed the impact OS can have on inclusivity, and how that can contribute to culture change:

**“The effect of open science on research is immense but also imperative, in my opinion. Inclusivity, intentful[sic] work, and collaboration would flourish, de-emphasizing the culture of competition and hyper-productivity that permeates academia.”**

## DISCUSSION

### OS Curriculum Integration Review

After its first implementation, the OS curriculum was deemed a success by the faculty instructor, FYRE program coordinator, and the University Library in that it provided an opportunity for students to learn about and practice OS, fostered student engagement in OS principles and practices, and demonstrated that students understood the value of practicing OS in a research context. The faculty instructor noted specifically that the integration led to students having a stronger sense of community and willingness to collaborate compared to previous years, a genuine interest in sharing their research products and seeing those of their classmates, and a sincere enthusiasm for OS at the end of the course. Finally, this work confirmed that integrating OS into curricula could be achieved through a librarian-led collaboration.

One of the perceived challenges before implementing the OS curriculum was that the combination of learning about OS, having to create unfamiliar research outputs like data dictionaries or data analysis plans, and using new software to manage their research would overwhelm students at this early stage of their academic career. However, our experience revealed that students grasped OS concepts quickly, were able to create research outputs with relative ease, and were comfortable making their research outputs public on the OSF platform.

When applying OS principles via the checkpoints, students struggled in two areas. First, students often failed to include their names on each research output; it had to be communicated to them multiple times through feedback that even though their research outputs were stored within an attributed OSF project, that if someone were to download and use an individual output for their own research, they would not know how to attribute the work. Second, some students struggled with making their research outputs interpretable by others outside of their immediate teams. Students frequently had to be reminded that sharing their research publicly meant they had to attempt to make the contents of each output as clear and interpretable as possible for lay users. One common example of this challenge was that students would treat each research output in isolation without any context as to how it related to their broader research project. To address this issue, structured feedback was provided asking students to provide introductory contextual statements at the beginning of each research output to explain its relation to the broader study, and link to the research outputs on OSF created in previous checkpoints so a lay user could explore all related research material. Examples from the librarian's own research projects were presented during the in-class feedback sessions to illustrate how to connect an individual research output to a broader project and link to other research outputs using OSF. After receiving this feedback, we noted that the interpretability and transparency of student research outputs improved significantly in OS checkpoints 3, 4, and 5 (Figure 1). All teams fulfilled the requirements for each checkpoint, with an average of two of the seven teams being asked to resubmit per checkpoint based on the challenges highlighted above.

### Transferability to Other Institutions

We believe that the OS curriculum and its accompanying materials presented in this paper could be adapted to other undergraduate or graduate student contexts where a research project is required. With respect to the time required to implement this curriculum, we have outlined the number of hours needed to complete each component in Table 1.

**Table 1:** Time commitment required for OS curriculum integration.

Open science curricular component	Instructor	Librarian
Writing and discussing curriculum proposal	3 hours	3 hours
Creation of syllabus	2 hours	2 hours
Scheduling Open Science Integration	2 hours	2 hours
Developing Introduction to Open Science Class	N/A	8 hours
Developing Open Science Checkpoint Content	N/A	8 hours
Introduction to Open Science Class	N/A	1.5 hours
Open science checkpoint feedback sessions	N/A	1.5 hours
Evaluation of open science checkpoints	N/A	8 hours
Responding to student questions via email	N/A	2 hours
Evaluating Reflection Assignments	10 hours	N/A
**Total hours per role:**	**17 hours**	**36 hours**

From the perspective of sustainability, we encourage libraries to upskill liaison librarians in OS to allow the curriculum to be taught broadly across an institution or to evenly distribute the responsibility of providing the course on a yearly basis. As an additional time saving option, we believe the Introduction to Open Science class could be pre-recorded and serve as required viewing for students as an alternative to being taught synchronously. This viewing could be followed up by a librarian providing a shorter synchronous overview at the beginning of the course to highlight specific OS topics and respond to student questions. The curriculum could also be modularized to enable the use of select OS checkpoints that suit a given research project. Finally, we encourage libraries with institutional or data repositories to use these platforms instead of OSF. We believe this curriculum could increase the early adoption and use of repository platforms by having students use them in the context of their own coursework and research.

If this course were to be taught to a general audience rather than integrated into a curriculum, it could be offered as a self-guided open educational resource and marketed to students who are in the initial stages of developing a research project. While they would not receive the same level of feedback as they would from having the material taught in the context of their course content, it would still provide an opportunity for them to practice OS at every stage of the research process.

### Outcomes and Next Steps

The OS curriculum will continue to be offered through the Nutrition course and its success has led the authors of this paper to explore opportunities to expand OS training to other disciplines within the FYRE program, particularly the Geography & Planning department. One key challenge the library has identified moving forward is the need to upskill librarians in other disciplines to be able to integrate the OS curriculum more broadly. The success of this first integration with Nutrition has created the impetus for the library to invest the time and resources into doing so.

To evaluate whether this curriculum will have a long-term impact on students' OS practices, we aim to follow up with the Nutrition students who took this course when they enter the final year of the undergraduate program to re-assess their OS engagement. Because much of the Nutrition program focuses on professional practice, there will be several opportunities for students to apply OS principles to future class projects as they progress through the program.

This project has also led to increased exposure of OS training on campus and has been a catalyst for providing new standalone workshops at the undergraduate and graduate level. These workshops have featured in the University's Summer Undergraduate Research Experience program (2021-2022) which is offered to students working on summer research projects with faculty on campus, within graduate and postdoctoral seminar courses in the College of Nursing and the College of Pharmacy and Nutrition (2020-2022), and in a recurring library workshop series offered broadly to students, faculty, and staff across the institution (2019-2022).

Increasing calls from government, funders, and publishers for researchers to adopt OS practices creates an opportunity for librarians to fill a training need in this area. Introducing OS principles to students when they are first exposed to the research process can make a strong impression on how they conduct research in that they are not entrenched in existing research practices and are therefore more receptive to an open approach. We see benefit in incorporating this curriculum into undergraduate and graduation education to encourage students to adopt OS principles early in their research careers and believe health sciences libraries are well positioned to take on this role.

## LIMITATIONS

This study provides one example of integrating OS into an undergraduate professional practice course, and the results found here may not be representative of all students participating in undergraduate professional practice courses. Similarly, the amount of time devoted to this project is indicative of one librarian and one faculty member's ability to carry out the work, and may vary depending on the work style, workload, and capacity of other librarians and faculty who may attempt to replicate this curriculum integration.

## Data Availability

Student reflection assignments cannot be shared because students did not consent to having their full assignment shared publicly. All materials from this course including the written proposal, FYRE program instructions, presentation slides, assignment templates, and aggregate data with the key themes that emerged from the reflection assignments, including codebook, are available as supplementary files.

## SUPPLEMENTAL FILES

**Appendix A:** FYRE OS Proposal to Faculty

**Appendix B:** Class Slides

**Appendix C:** Course Instructions

**Appendix D:** Data Dictionary Template

**Appendix E:** Reflection Assignment

**Appendix F:** Aggregate Data and Codebook
