# Supplementary material for: Integrating open science education into an undergraduate health professional research program
Source: J Med Libr Assoc. 2022 Oct 1;110(4):429–37. doi: 10.5195/jmla.2022.1457 (PMC10124608; doi:10.5195/jmla.2022.1457)
Supplement: Supplementary file 1 — Appendix A: FYRE OS Proposal to Faculty [file jmla-110-4-429-s01.pdf]

Integrating Open Science Education into the FYRE Program: A Pilot Study
Study Outline

Appendix A: FYRE OS Proposal to Faculty

Potential Investigators:

[include names here]

Background:

Open science (OS) has been defined as the practice of making everything in the research process fully and openly available, creating transparency, and driving discovery by allowing others to build on existing research. Over the last few years, OS has become a global phenomenon geared towards promoting transparency and reproducibility in research and facilitating secondary research. With the current pandemic accelerating research at a rapid rate, studies highlighting concerns over published research articles that cannot be reproduced, and an increasing spotlight on how published research does not provide sufficient detail in their protocols and methods, OS serves to improve the transparency of the research process from inception to publication.

Objective:

Currently, there are very few examples of OS educational initiatives in academia. While elements of OS like scholarly communication and research data management education have become more common, these trainings are almost entirely geared towards graduate students, and do not present concepts within the lens of OS across the research lifecycle.

The FYRE program presents a unique opportunity to educate undergraduate students about OS at the very beginning of their introduction to research. Using NUTR 230 as a pilot, this project proposes to: a) develop an educational intervention to introduce undergraduate students to OS best practices and tools, b) apply OS best practices at each project checkpoint that can be assessed and evaluated, and c) capture students' views of OS at the completion of the project through interviews or a survey. Using these three steps, undergraduate students can learn about, apply, and reflect on OS during their first experience conducting research, while providing insight into whether this will contribute to them being more likely to apply OS best practices going forward.

Education Learning Objectives:

Students will be able to:

- Define open science and describe current challenges surrounding research transparency
- Outline current open science initiatives in research
- Describe processes used to make their research materials more openly available
- Communicate the value of applying open science best practices
- Apply open science best practices using the Open Science Framework (OSF) tool

Open Science Assessment:

This project proposes that students create an Open Science Framework instance of their project to establish research transparency. At each project checkpoint, students will be asked to consider their

research outputs and communicate these outputs openly using the Open Science Framework tool by uploading them to their project instance. Each checkpoint output is described in Table 1.

Integrating Open Science Education into the FYRE Program: A Pilot Study
Study Outline

Table 1.

**Student Milestone**	**Open Science Component**
Checkpoint 1:<br>Submission of Research Question and Justification	Students create a collaborative OSF project and upload the finalized research question and justification.
Checkpoint 2:<br>Submission of Survey Questions	Student create a data dictionary of their survey questions and upload to their OSF project.
Checkpoint 3:<br>Submission of Draft Introduction and Methods Section of the Poster	Students upload their introduction and methods to their OSF project.
Checkpoint 4:<br>Data Analysis Progress and Draft Poster	Students write a summary of the analysis procedures they have applied to the data and upload it to their OSF project.
Final product:<br>Completed poster	Students upload their completed poster to their OSF project.

Post-project Assessment/Reflection:

After the project is complete, students would either complete a survey or sit down for a group interview asking them to reflect on their experiences making their research more "open" throughout the research process. Questions would ask students about their views of open science – both positive and negative, whether they would consider applying open science practices in future research projects, and what value (if any) they see in open science practices being applied more widely across all research disciplines.

References

1. The Royal Society. Science as an open enterprise. London: The Royal Society; 2012.

2. Watson M. When will 'open science' become simply 'science'?. Genome biology. 2015 Dec;16(1):1-3.
