# Supplementary material for: Integrating open science education into an undergraduate health professional research program
Source: J Med Libr Assoc. 2022 Oct 1;110(4):429–37. doi: 10.5195/jmla.2022.1457 (PMC10124608; doi:10.5195/jmla.2022.1457)
Supplement: Supplementary file 2 — Appendix B: Class Slides [file jmla-110-4-429-s02.pdf]

University of Saskatchewan

Open Science in Practice

NUTR 230 | FYRE

CC BY NC

What do you associate with the word OPEN?

What do you associate with the word CLOSED?

University of Saskatchewan

Open

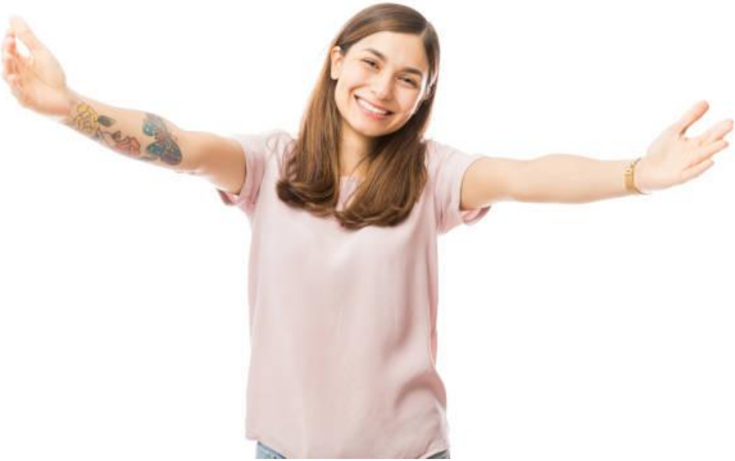

https://tinyurl.com/yxu8nw38

Closed

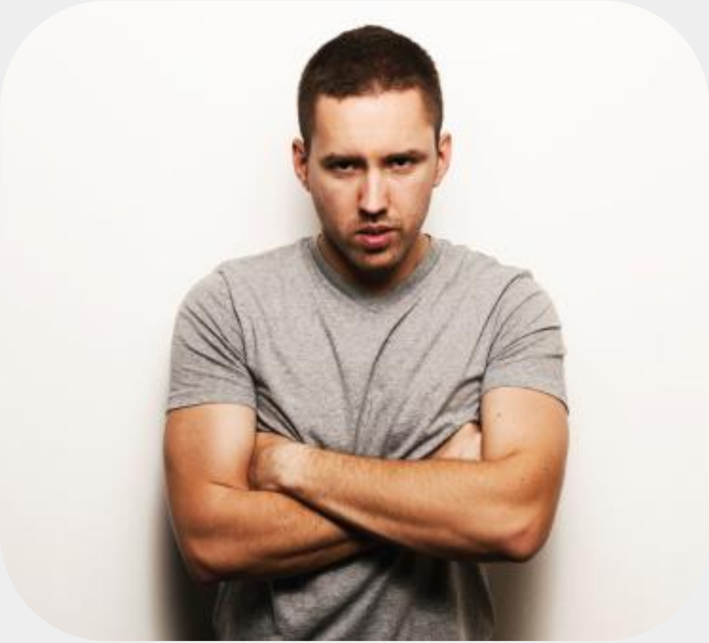

https://tinyurl.com/y2eq2ngl

Learning Objectives

Articulate the difference between closed and open science approaches

Define open science

Outline the different stages of the research lifecycle

Identify current open science initiatives

Apply best practices in open science

FYRE Lifecycle

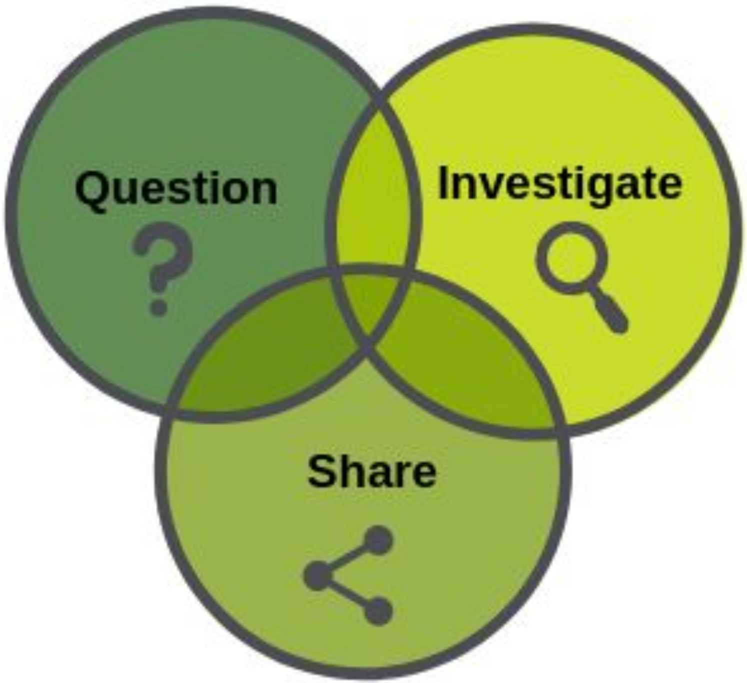

Research Lifecycle

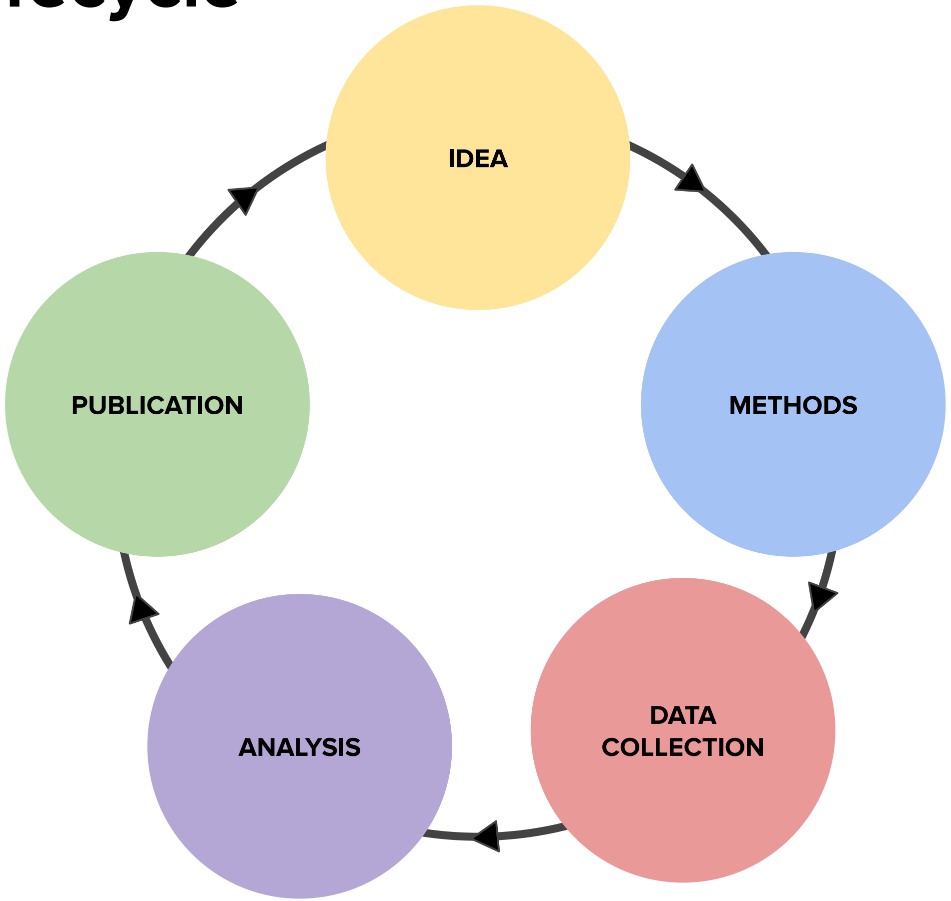

Research Lifecycle

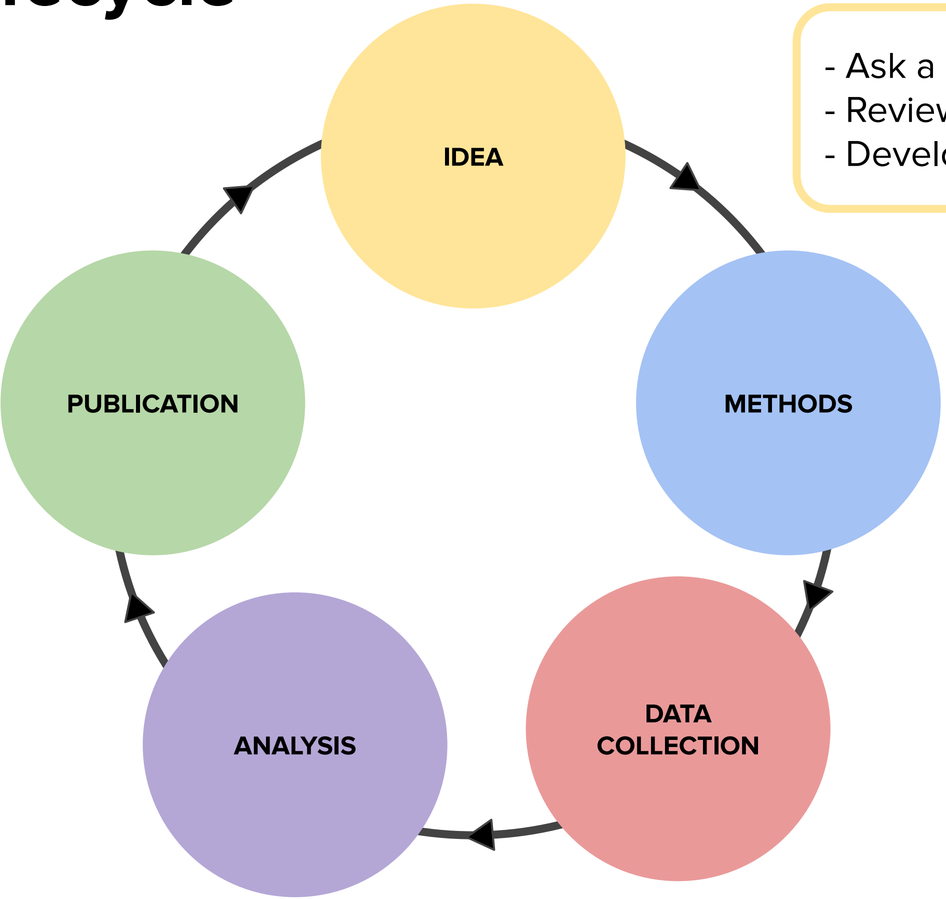

- Ask a research question
- Review existing research
- Develop hypothesis

University of Saskatchewan

Research Lifecycle

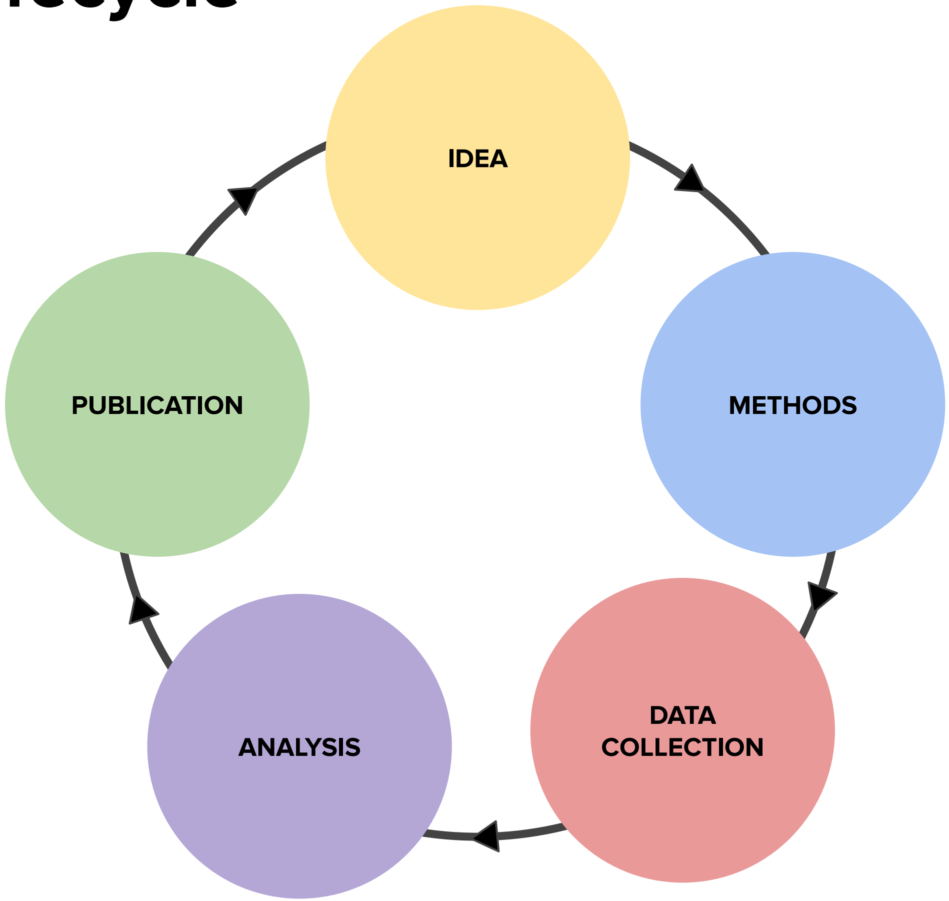

- Develop instruments
- Plan experiments
- Identify participants and/or subject(s)

Research Lifecycle

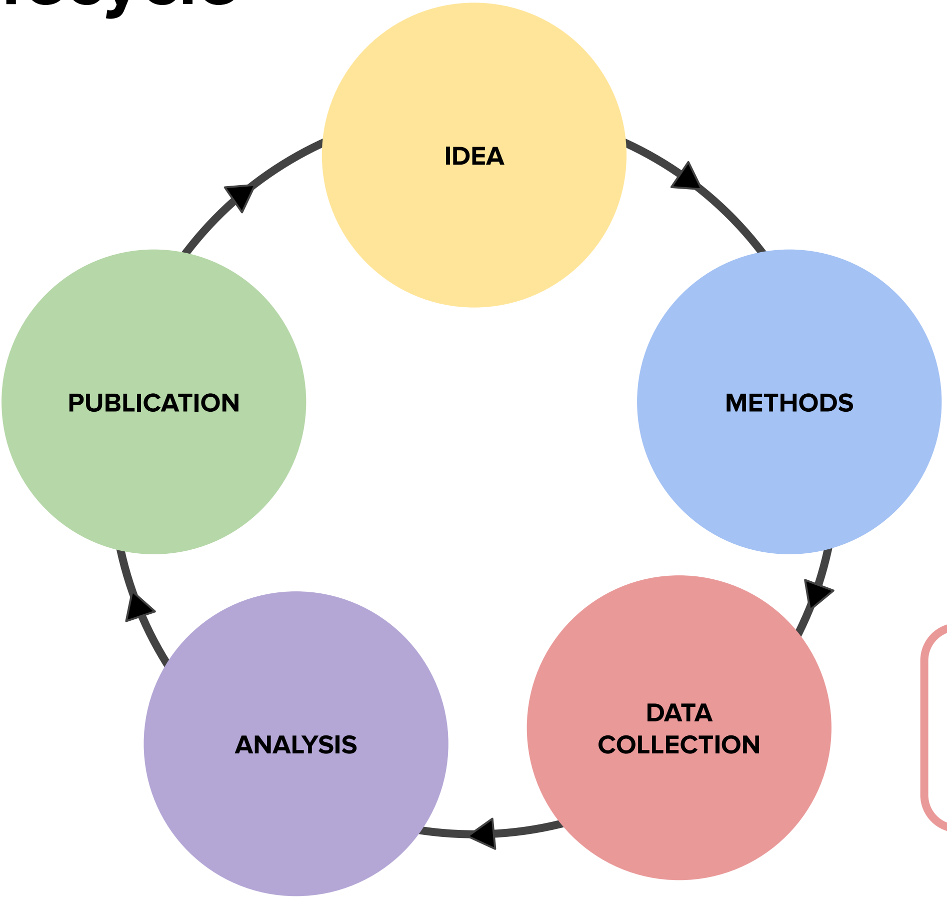

- Gather text, numbers, images, etc.
- Store data
- Describe data

Research Lifecycle

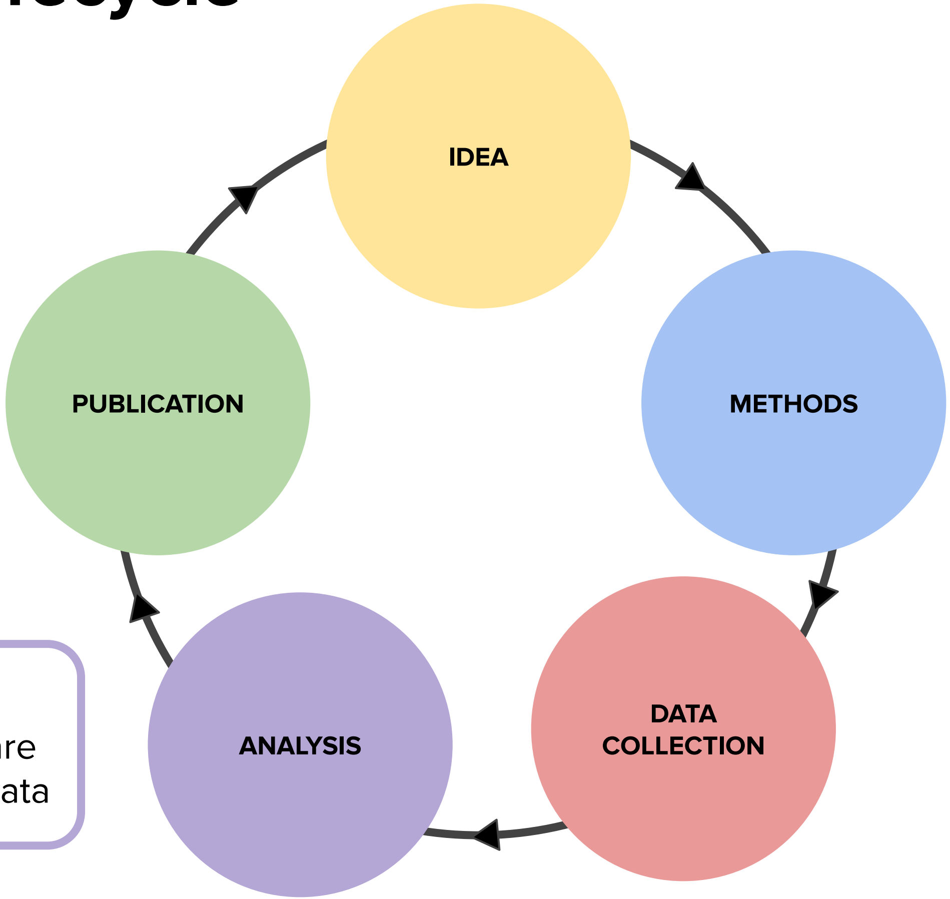

- Build analysis plan
- Use statistics software
- Analyze/transform data

Research Lifecycle

- Present at conference
- Publish in journal
- Share research data

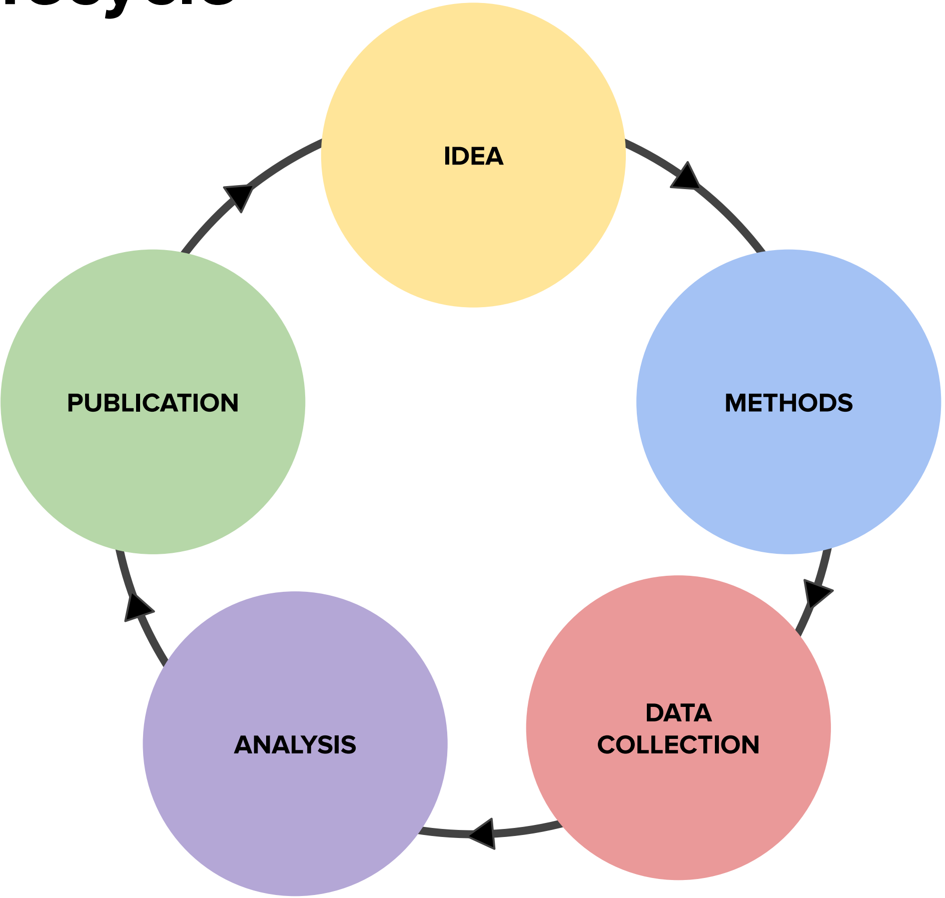

Scientific Research Today

Closed Science

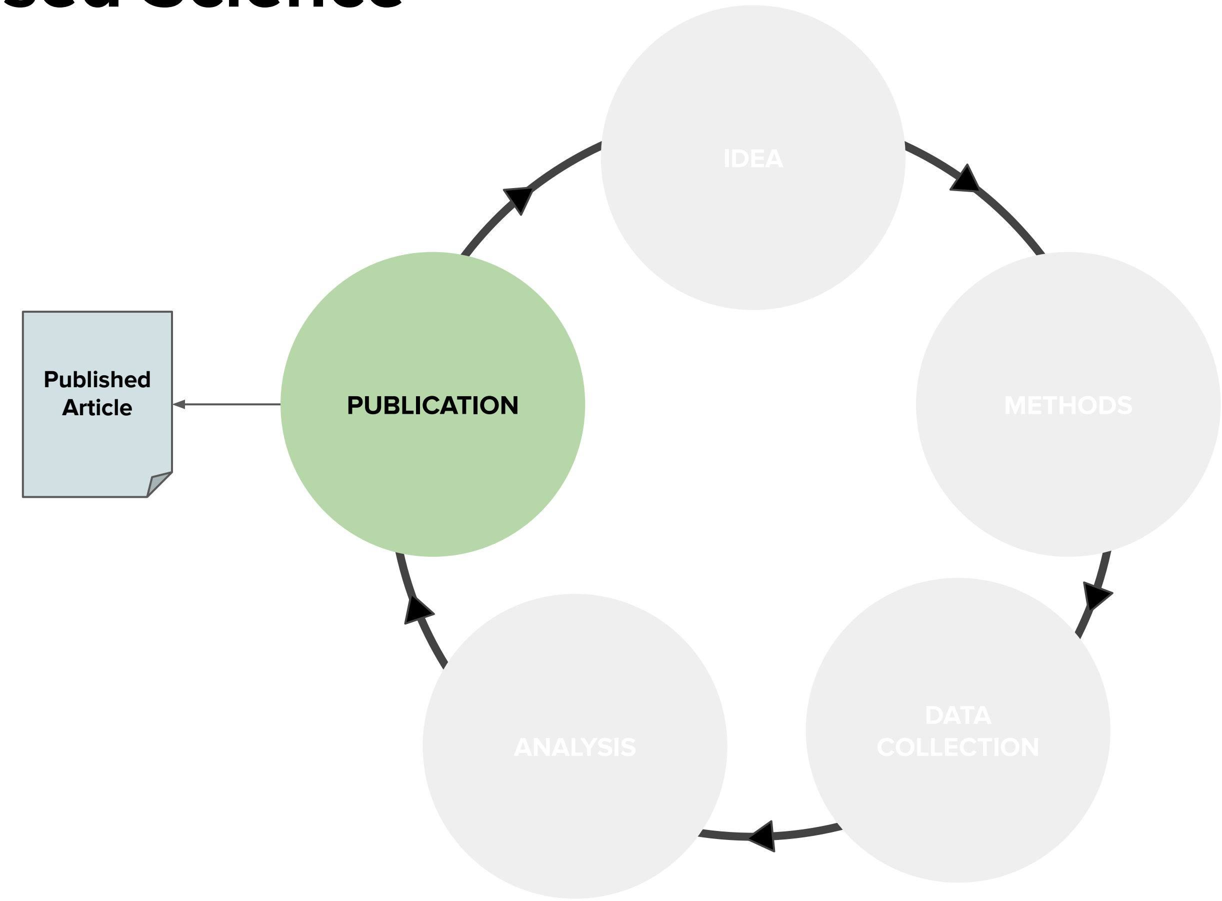

Closed Science

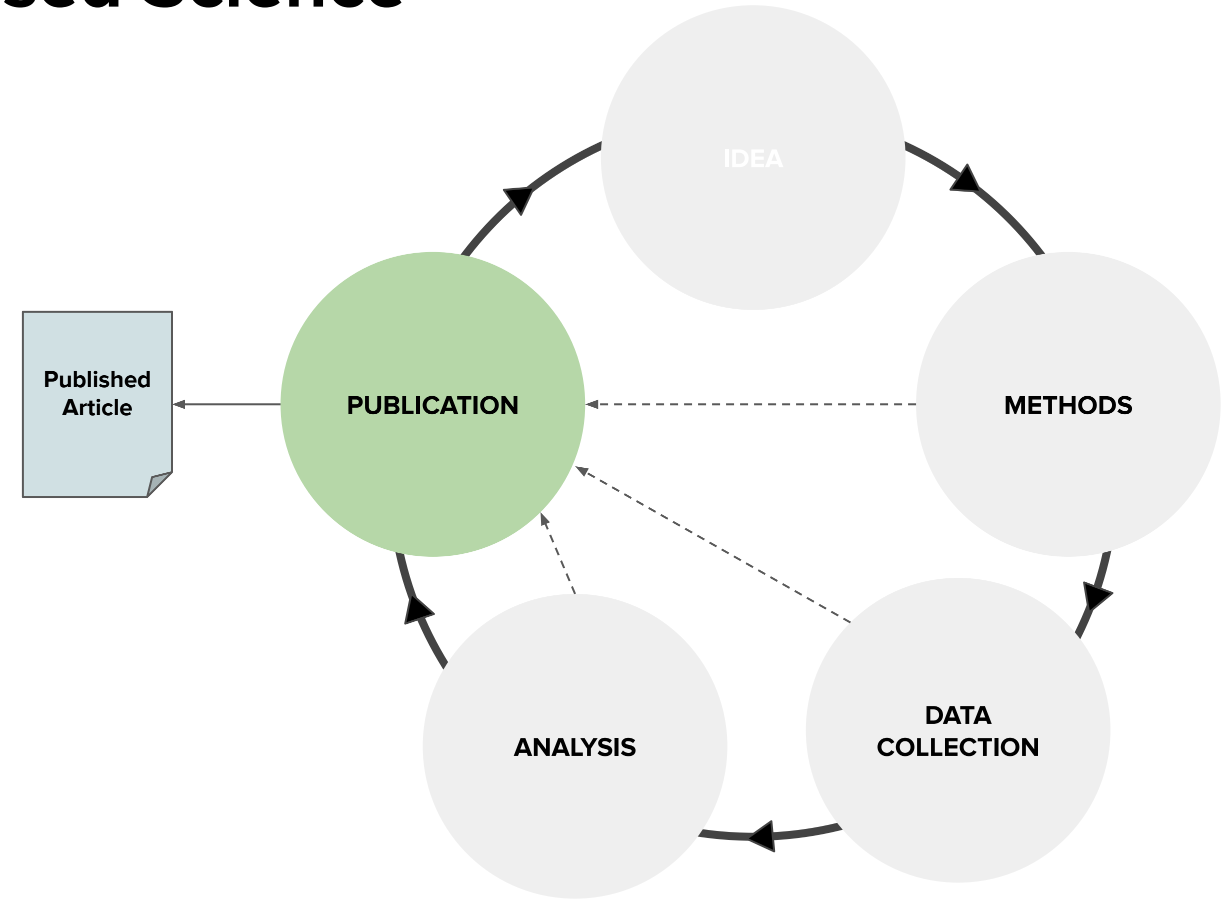

University of Saskatchewan

Publication / Poster / Presentation

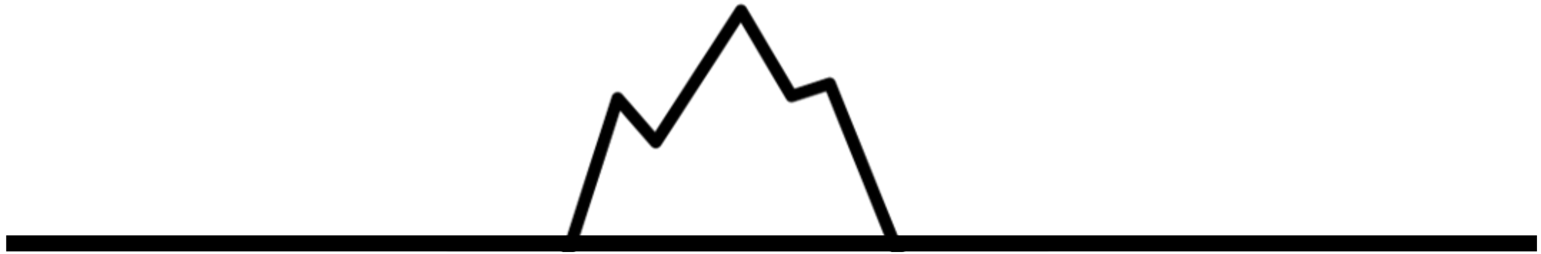

Why is Closed Science Problematic?

Hides key components of the research process

Makes reproducing or reusing research results difficult

Restricts access to research

Eliminates trust in research

Creates competitive atmosphere where researchers are pitted against one another

Reproducibility Crisis

What is reproducibility?
The ability to follow or implement the same experiments using the same data/tools to get the same results

Recent studies have found that research results ***could not*** be recreated

Common issues:

- Lack of transparency
- Poor documentation

http://www.nature.com/news/over-half-of-psychology-studies-fail-reproducibility-test-1.18248

NATURE | NEWS

Over half of psychology studies fail reproducibility test

Largest replication study to date casts doubt on many published positive results.

SundayReview

Why Do So Many Studies Fail to Replicate?

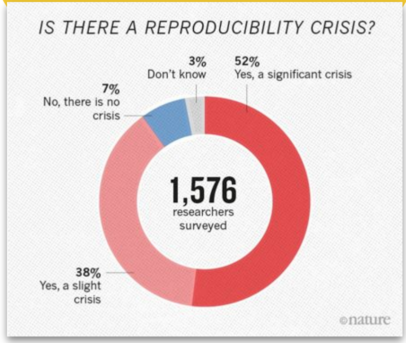

http://www.nature.com/news/1-500-scientists-lift-the-lid-on-reproducibility-1.19970

University of Saskatchewan

Closed Science

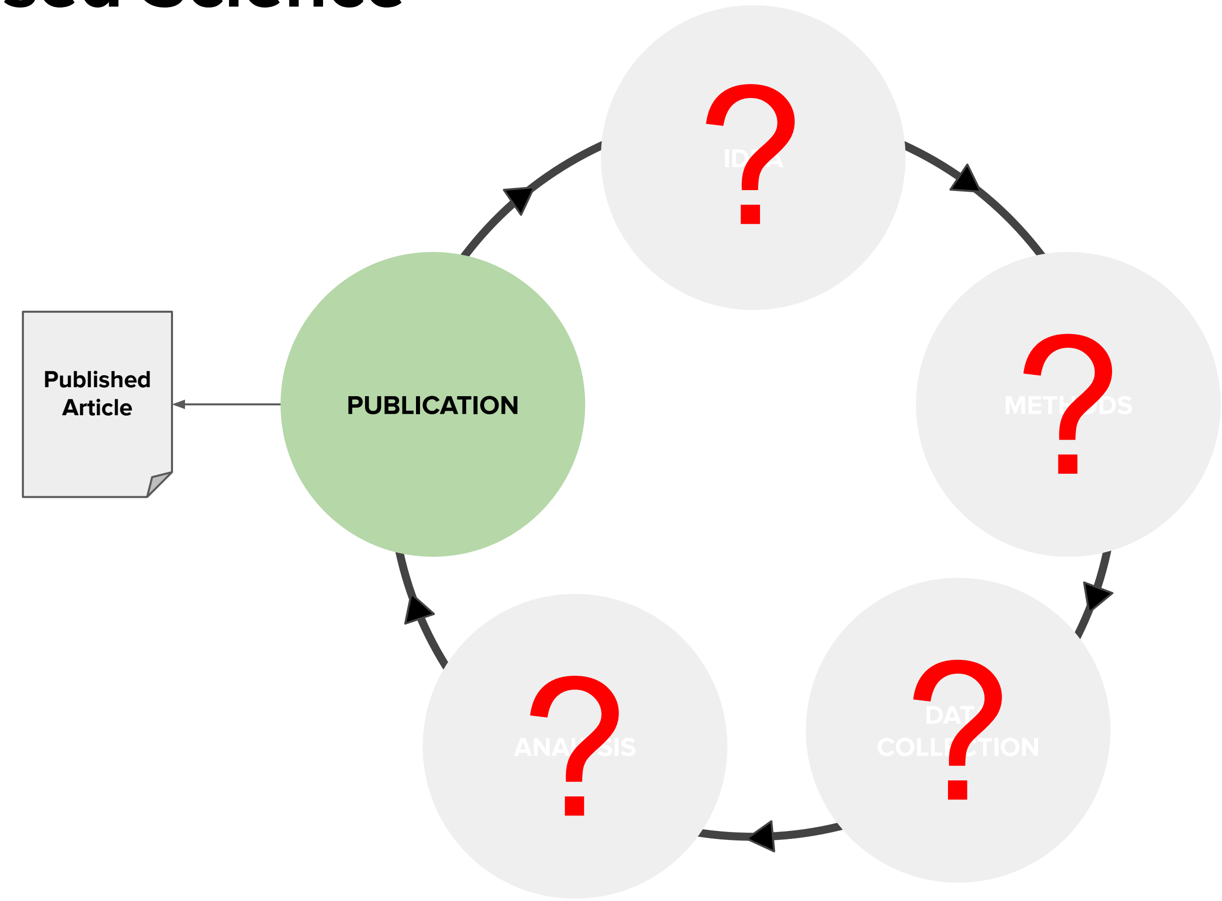

UNIVERSITY OF SASKATCHEWAN

An Example

HEAVENLY PIE

- 2 ripe bananas
- ⅛ t. salt
- ½ t. vanilla
- ¼ c. chopped nuts
- 1 c. granulated sugar
- 2 egg whites
- ½ pint whipped cream

Put in shell, put cream and chopped nuts on top.

MRS. R. D. SICKAFOOSE, Magnolia Grange, Stark County

http://1.bp.blogspot.com/-vHN5EzfAmBA/Vbesj-Aq12I/AAAAAAAACq8/C1bQmrJaKq4/s1600/heavenly%2Bpie.png

The Research Process

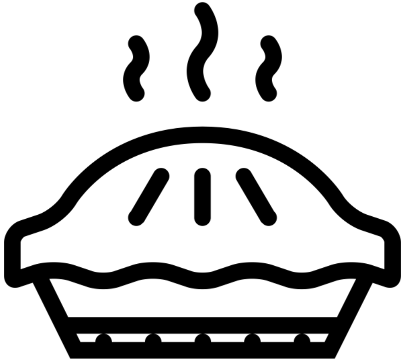

Heavenly Pie

=

Published journal article

Closed Science: Hidden process

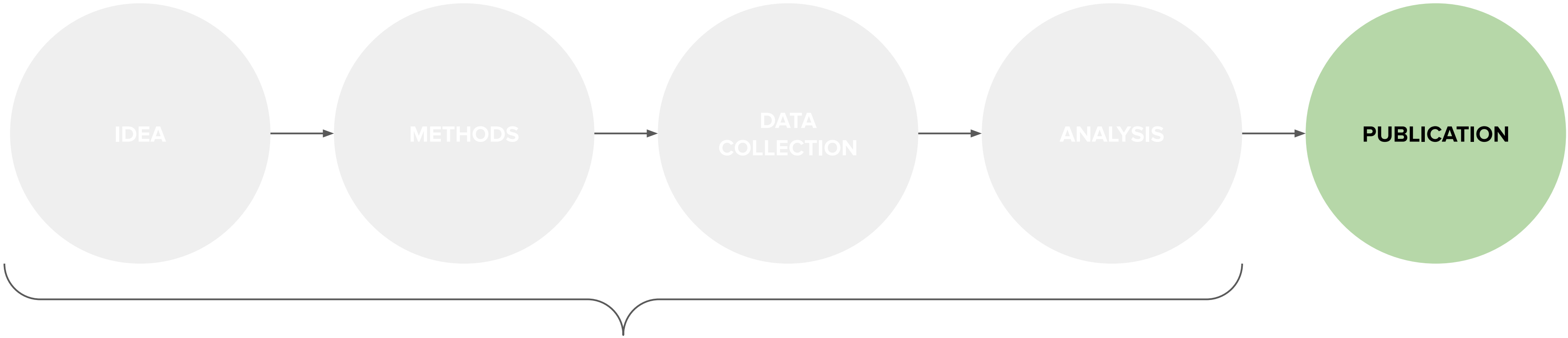

Peer review?
Transparency?
Trust?

Publishing articles: *Subscription Model*

Forces the public to pay for research that may have been publicly funded

Increasing expense of these journals make them difficult to access

Limits access to those who can afford to pay

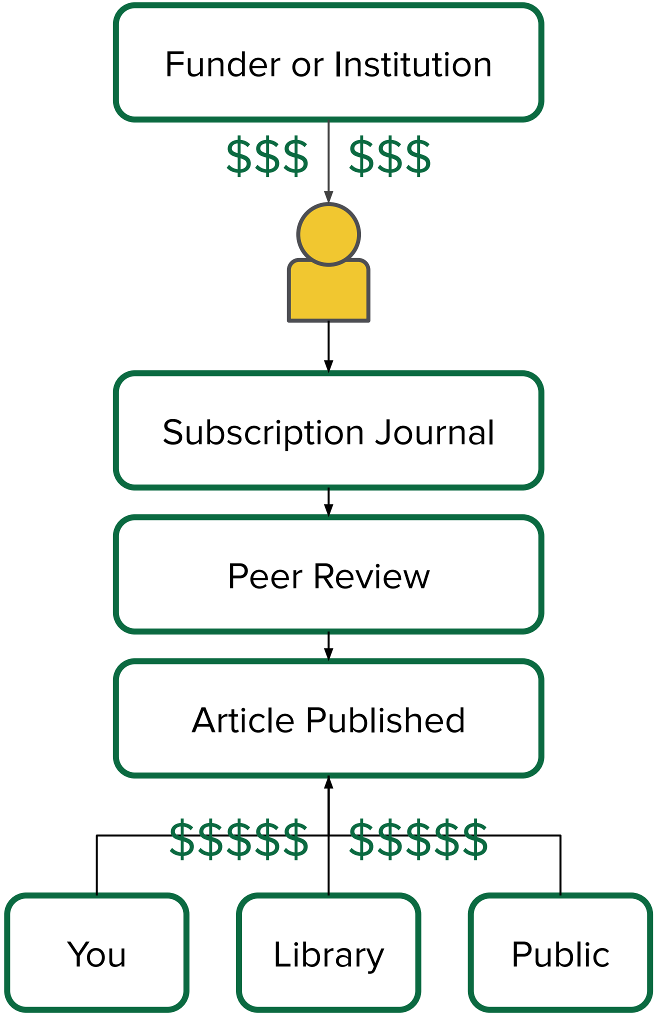

University of Saskatchewan

University of Saskatchewan

Closed Science: Subscription Model

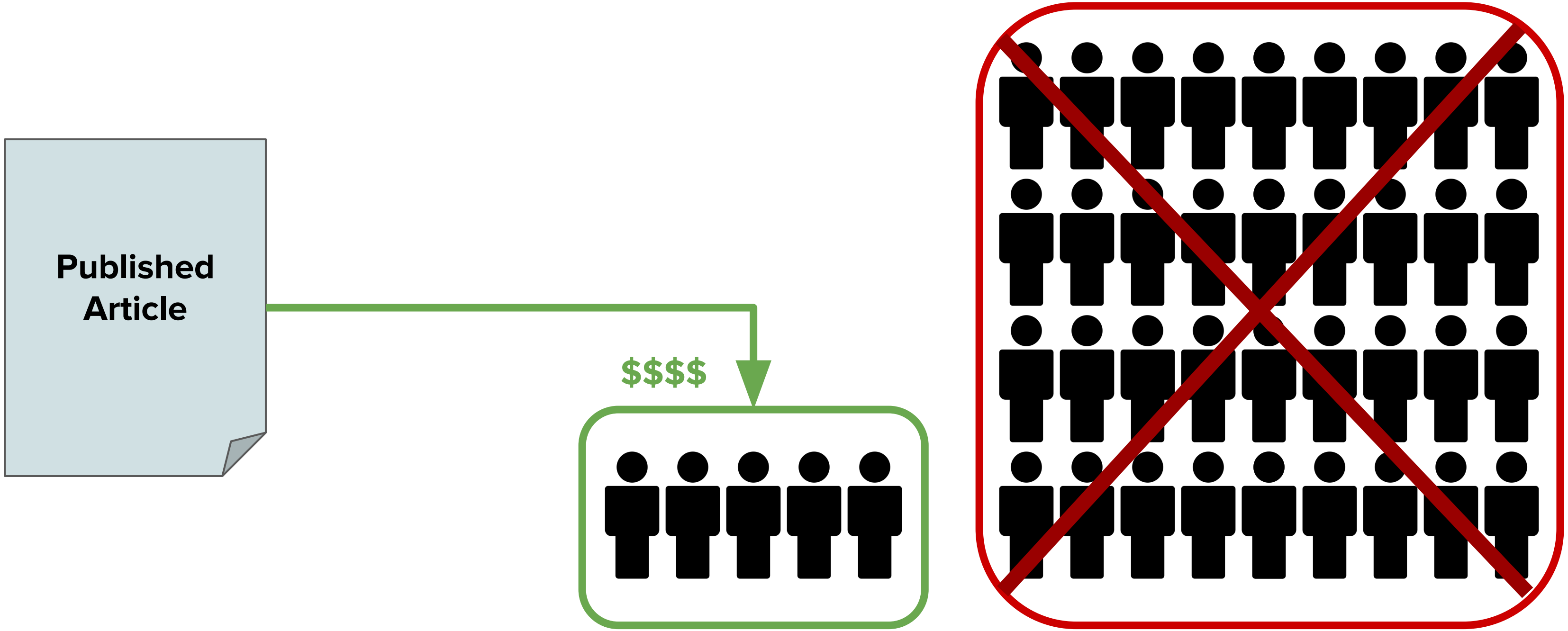

University of Saskatchewan

Closed Science

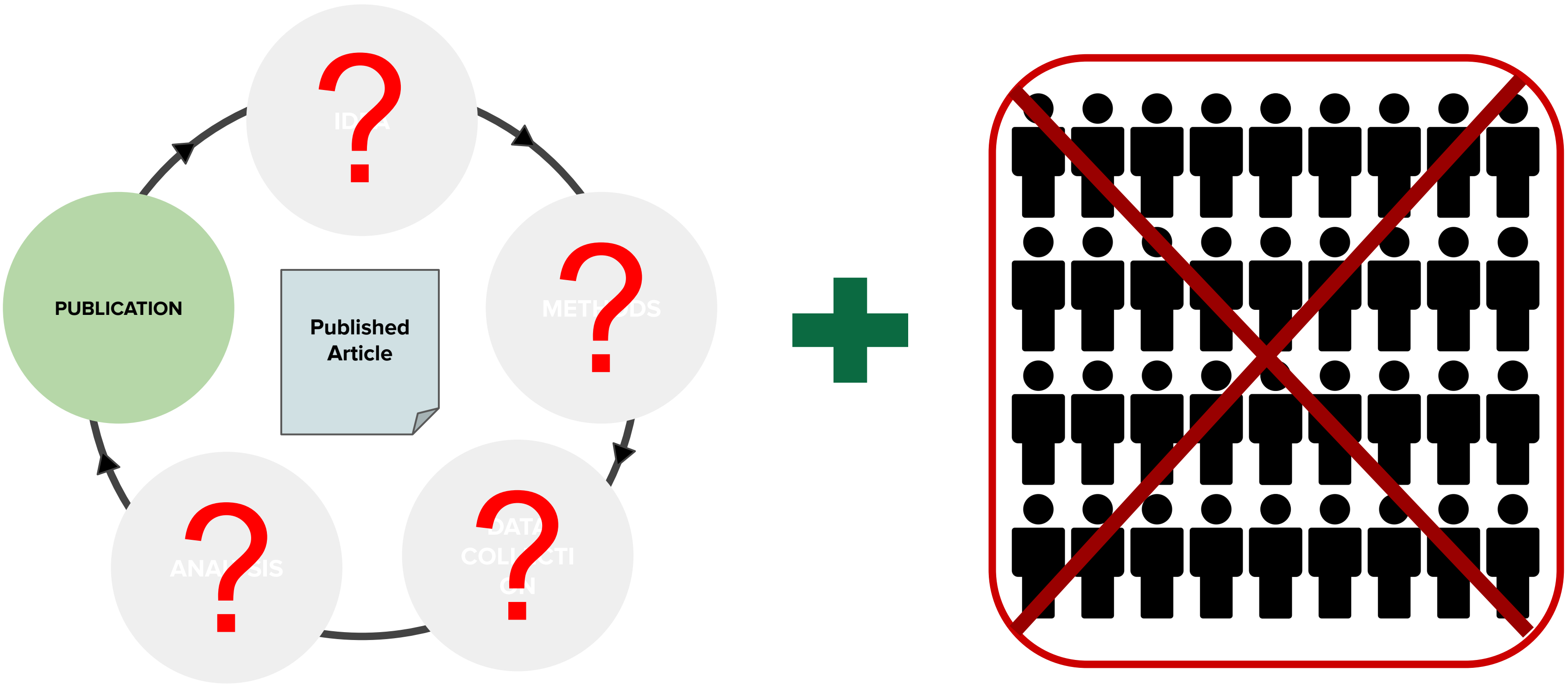

Transitioning to Open Science

What is Open Science?

Goals:

1. To make the products (e.g., publication, data, methods) of publicly funded research results publicly accessible with no or minimal restriction (OECD, 2015)
2. Foster sharing and collaboration as early as possible in the research process
3. Creating a systemic change to the way science and research is done

University of Saskatchewan

Open Science

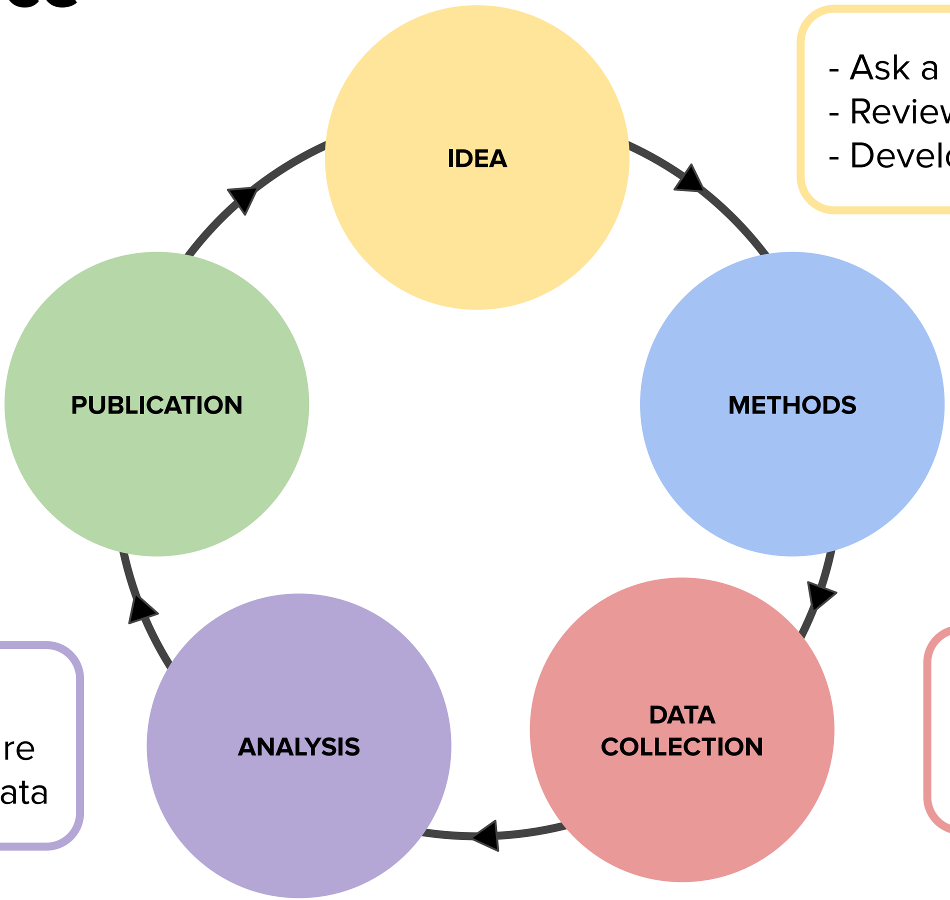

- Ask a research question
- Review existing research
- Develop hypothesis

- Develop instruments
- Create experiments
- Identify participants and/or subject(s)

- Gather text, numbers, images, etc.
- Store data
- Describe data

- Build analysis plan
- Use statistics software
- Analyze/transform data

- Present at conference
- Publish in journal
- Share research data

CLOSED

University of Saskatchewan

Publication / Poster / Presentation

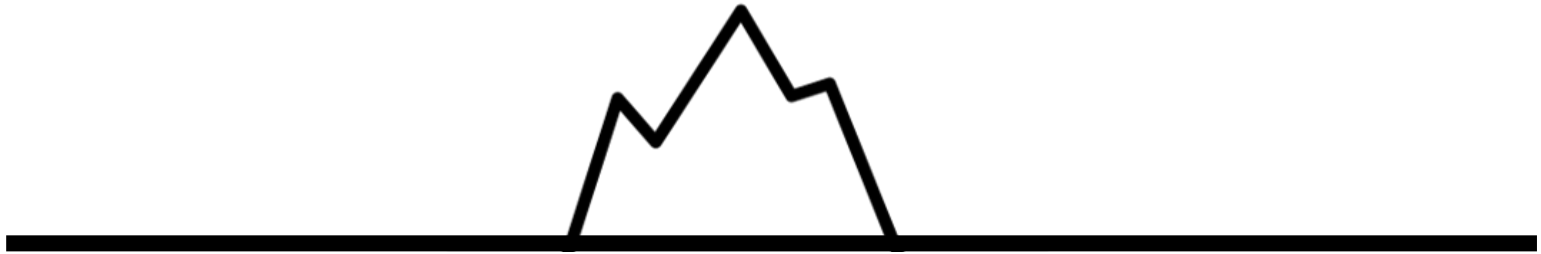

OPEN

University of Saskatchewan

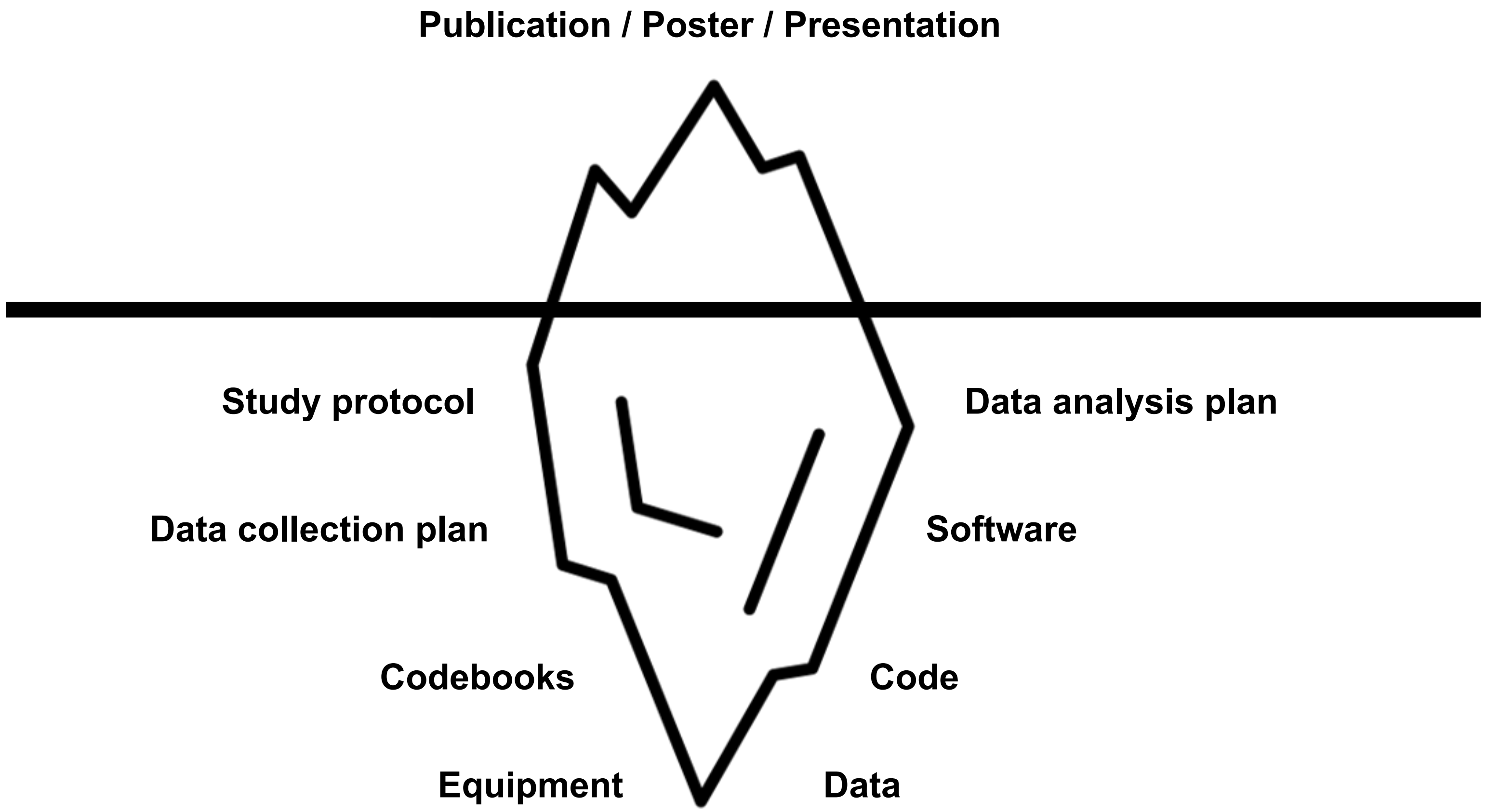

What are the Benefits of Open Science?

Improves the quality, integrity, and transparency of research

Increases efficiency in research

Research is openly available to all

Stronger engagement with the public

Increases collaboration opportunities = New/faster research

University of Saskatchewan

Our example revisited

HEAVENLY PIE

2 ripe bananas
⅛ t. salt
½ t. vanilla
¼ c. chopped nuts
1 c. granulated sugar
2 egg whites
½ pint whipped cream

Put in shell, put cream and chopped nuts on top.

MRS. R. D. SICKAFOOSE, Magnolia Grange, Stark County

http://1.bp.blogspot.com/-vHN5EzfAmBA/Vbesj-Aq12I/AAAAAAAACq8/C1bQmrJaKq4/s1600/heavenly%2Bpie.png

The Closed Research Process

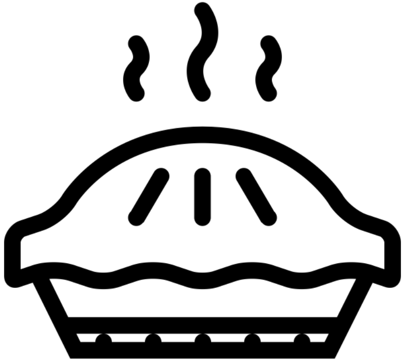

Heavenly Pie

=

Published journal article

An Open Research Process

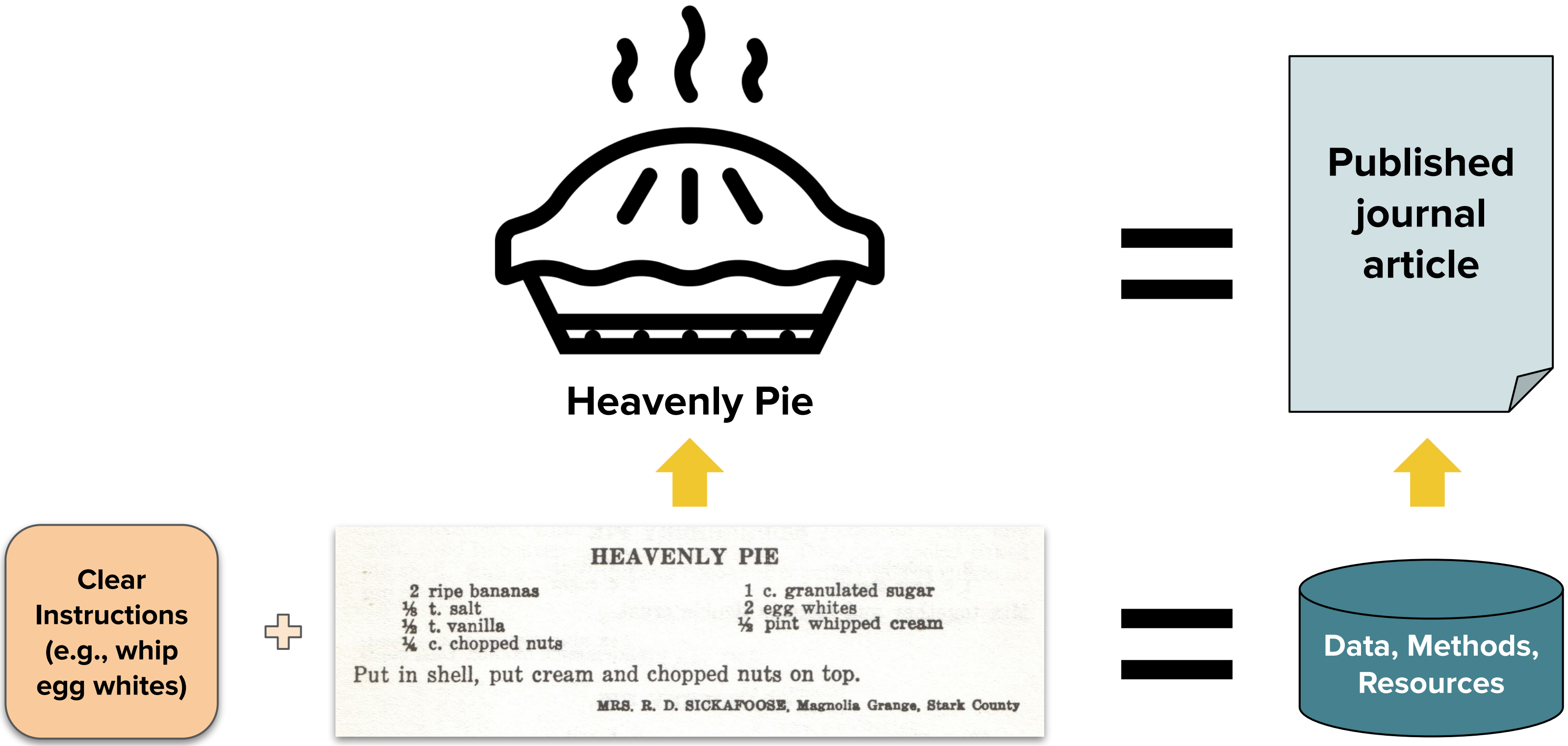

University of Saskatchewan

Open Science: Transparency is key

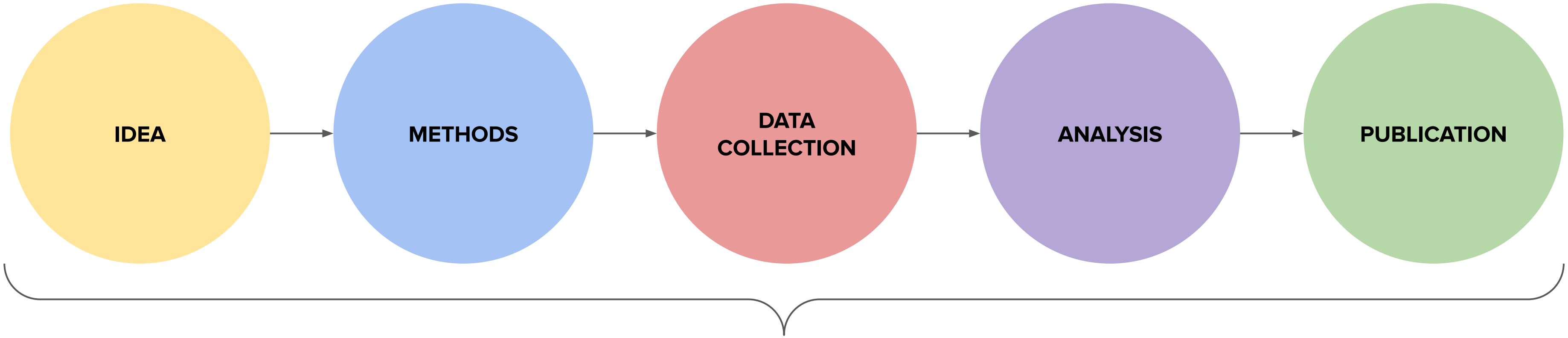

Clear instructions on how work is done

Opportunity for peer review at any stage

Access to products of research at each stage

Publishing: ***Open Access***

Research publications are available and accessible by anyone

Usually free of copyright so can be reused by others

Are cited more often that subscription based journals

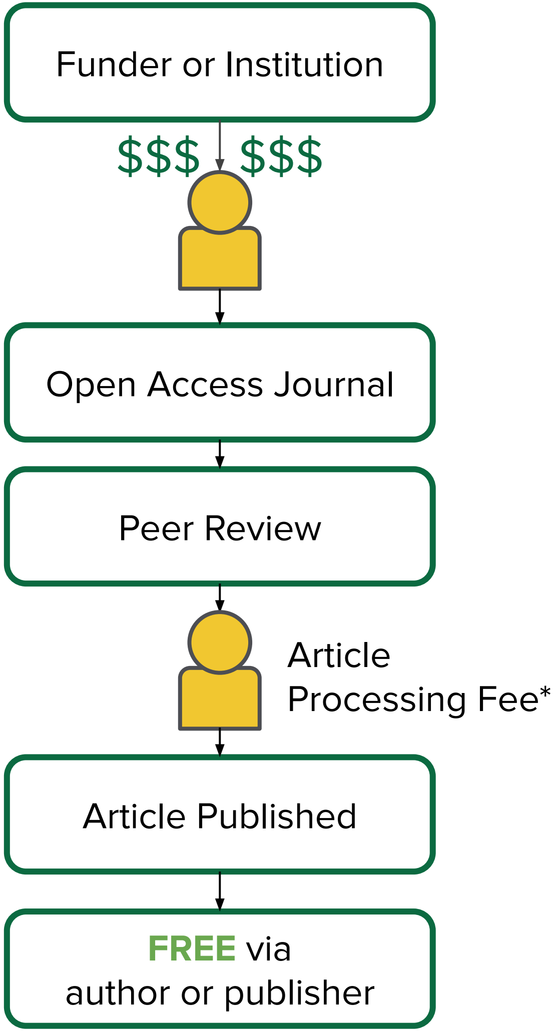

UNIVERSITY OF SASKATCHEWAN

Open Science: Open Access Model

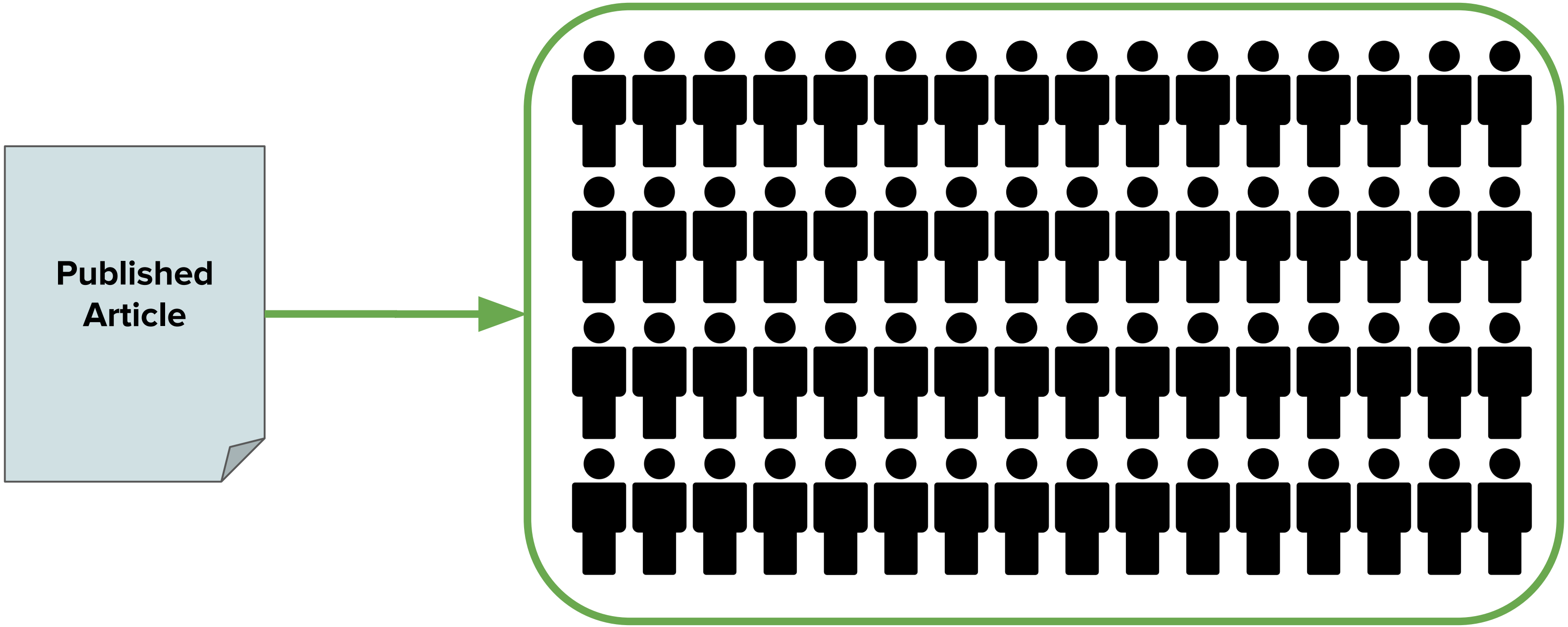

University of Saskatchewan

What does open science look like in action?

Covid-19 Research

https://www.nature.com/articles/d41586-020-01246-3

TECHNOLOGY FEATURE · 24 APRIL 2020

Open science takes on the coronavirus pandemic

Data sharing, open-source designs for medical equipment, and hobbyists are all being harnessed to combat COVID-19.

Mark Zastrow

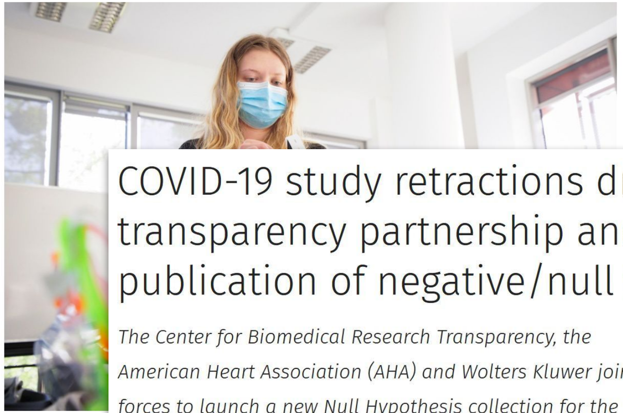

The key to finding a cure for COVID-19? Open science

GUY ROULEAU
CONTRIBUTED TO THE GLOBE AND MAIL
PUBLISHED JULY 10, 2020

6 COMMENTS SHARE

00:00 Voice 1x

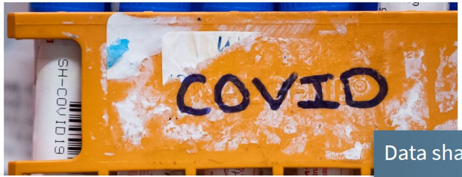

TRENDING

1 Trudeau cautions against extremism in John A. Macdonald statue debate

2 Over 65? Pay attention to your protein intake
LESLIE BECK

3 Gordon Pape: My growth portfolio is high risk, but it's generating incredible returns

4 Canada's bank regulator rolling back loan deferral programs for banks, insurers

https://www.theglobeandmail.com/opinion/article-the-key-to-finding-a-cure-for-covid-19-open-science/

COVID-19 study retractions drive research transparency partnership and push for increased publication of negative/null findings

The Center for Biomedical Research Transparency, the American Heart Association (AHA) and Wolters Kluwer join forces to launch a new Null Hypothesis collection for the AHA's scientific journals portfolio

CONTACTS

André Rebelo
Sr. Global Public Relations Manager

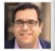

Health

Data sharing in the era of COVID-19

Christopher V Cosgriff · Daniel K Ebner · Leo Anthony Celi

Open Access · Published: May, 2020 · DOI: https://doi.org/10.1016/S2589-7500(20)30082-0

Severe acute respiratory syndrome coronavirus 2 (SARS-CoV-2) continues to test the capacity of world health systems. Since the outbreak started, the global community has learned about coronavirus disease 2019 (COVID-19), the disease resulting from SARS-CoV-2. In the first few weeks of the pandemic, knowledge about the disease and its treatment was generated from sharing of anecdotal observations and small case series. Although health-care professionals use modern technology to communicate, never before has the failure to build robust data-sharing systems for large-scale near real-time analysis in health care been more obvious.

In the era of electronic health records, physiologic, laboratory, imaging, decision-making, and treatment data are continuously recorded. Inferences drawn from these data can inform epidemiological inquiries and guide treatment protocols when clinical trial data do not exist or might be too slow to inform a rapidly evolving situation. While the number of trials increases, real-time treatment data accumulates, siloed within hospital systems. When considering COVID-19, the insight we could gain from a pooled, publicly available dataset analysed by researchers in academic institutes and industry is invaluable and necessary.

https://www.thelancet.com/journals/landig/article/PIIS2589-7500(20)30082-0/fulltext

https://www.wolterskluwer.com/en/news/covid-19-study-retractions-drive-research-transparency-partnership

UNIVERSITY OF SASKATCHEWAN

Montreal Neurological Institute

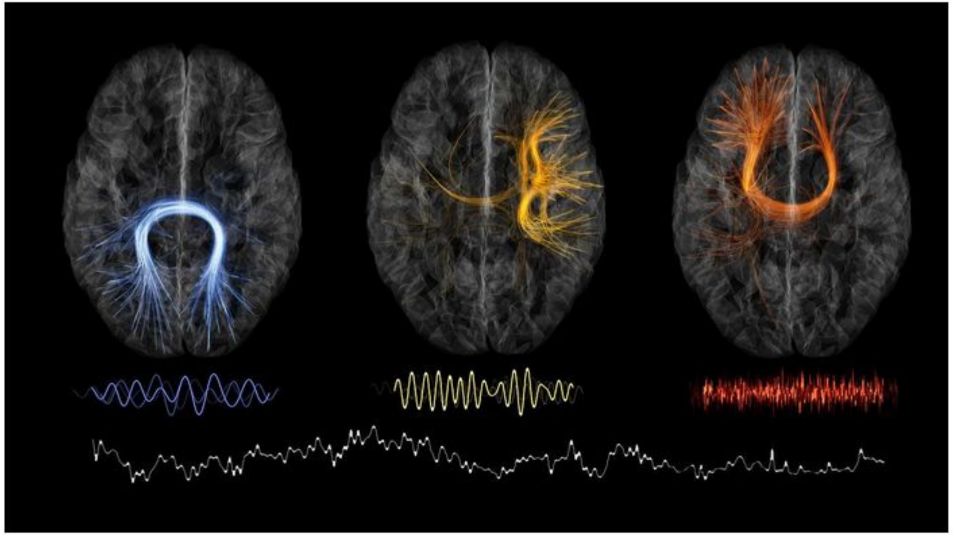

The Montreal Neurological Institute plans to free up its findings, including data that point to connections between brain regions communicating at different neural rhythms. SÉBASTIEN DERY, MCCONNELL BRAIN IMAGING CENTRE, MONTREAL NEUROLOGICAL INSTITUTE

Montreal institute going 'open' to accelerate science

By Brian Owens | Jan. 21, 2016 , 2:00 PM

https://www.sciencemag.org/news/2016/01/montreal-institute-going-open-accelerate-science

UNIVERSITY OF SASKATCHEWAN

Canada's Roadmap

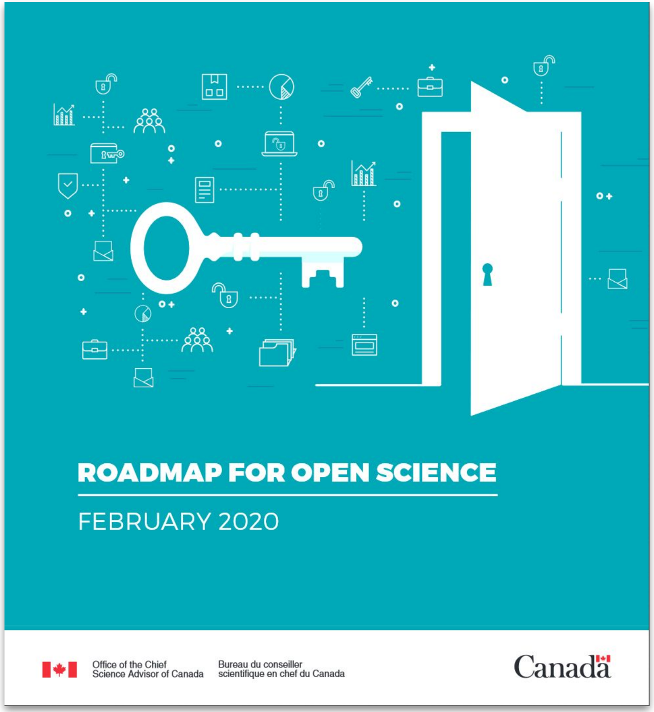

http://science.gc.ca/eic/site/063.nsf/eng/h_97992.html

"It will be required that federally-funded research publications, data, and materials be made openly available by January 2023."

Where to start with Open Science?

Throughout the research process, ask yourself:

How can I share what I'm doing with others from the very beginning of the process?

Can I hold myself accountable for what I've set out to do?

Will others be able to understand what I'm doing/have done?

Will my work be accessible to everyone?

University of Saskatchewan

Open Science

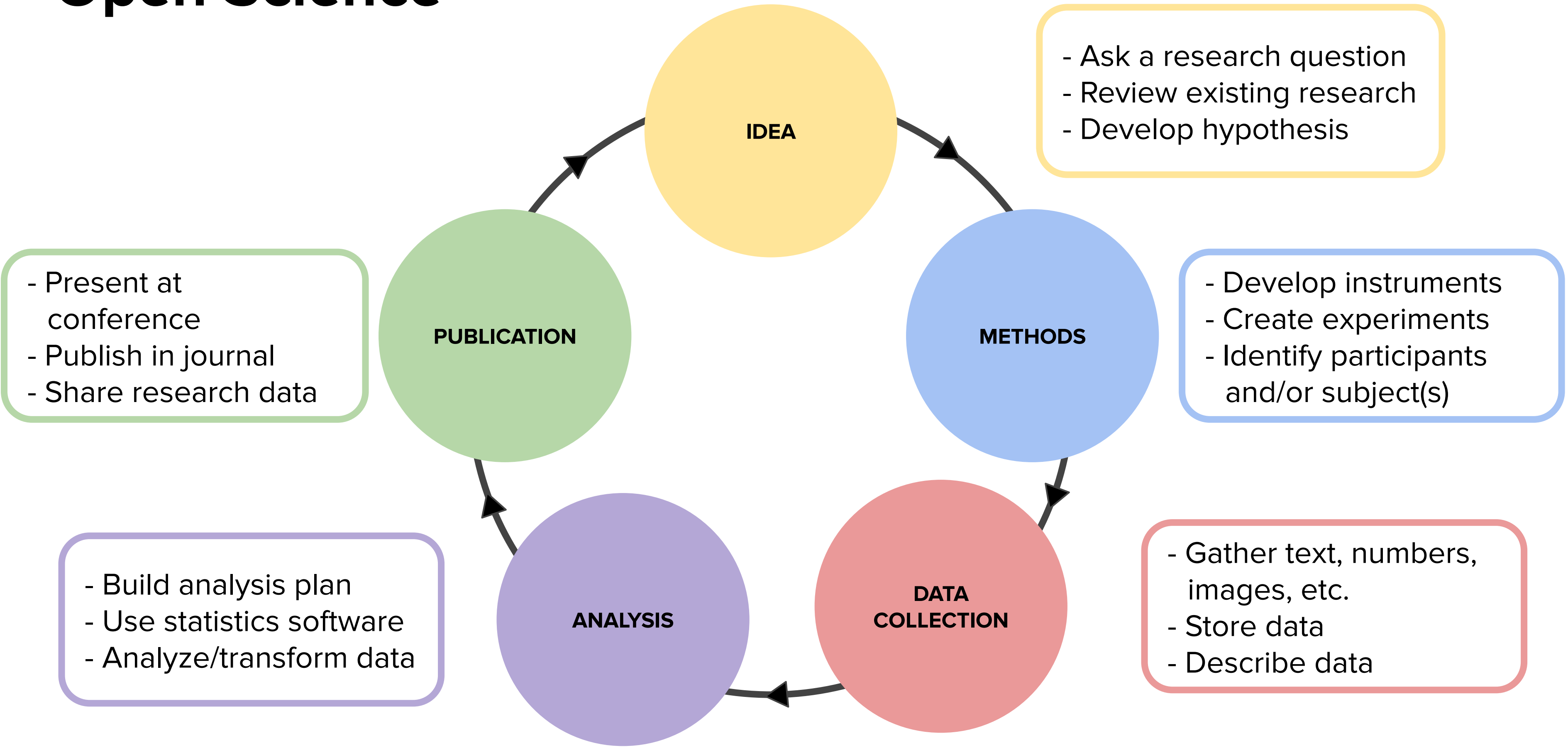

Open Science in Action

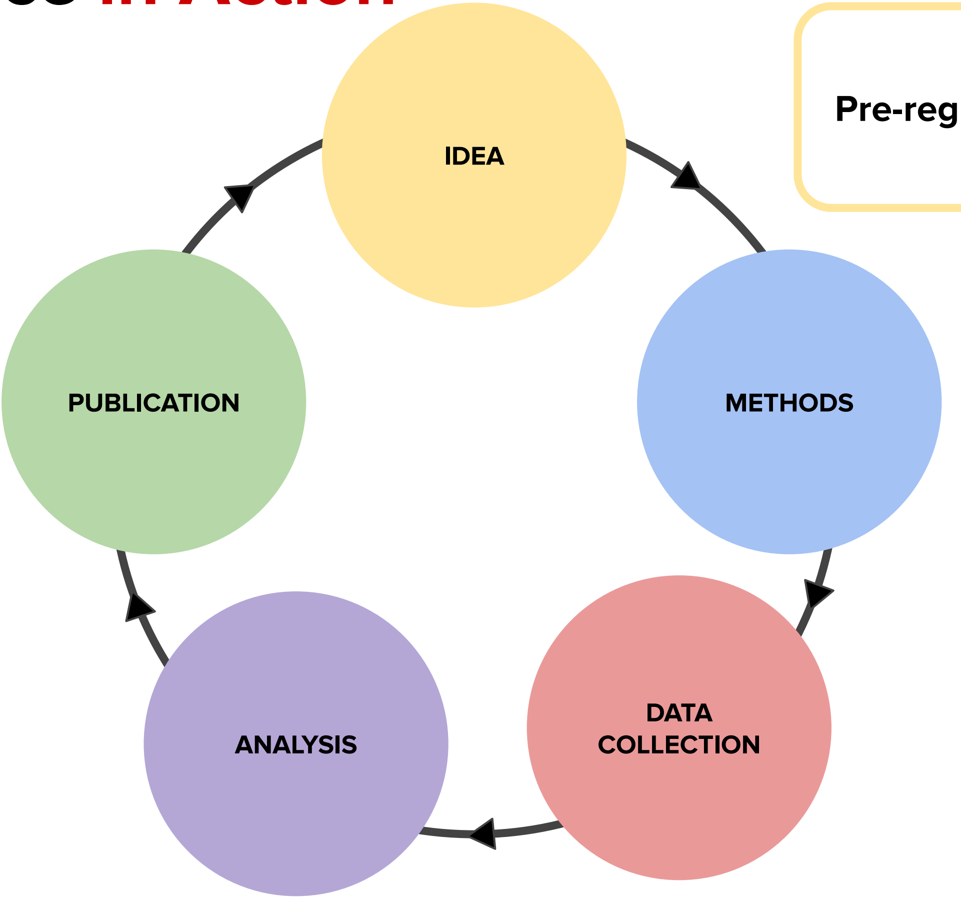

Pre-register your project

University of Saskatchewan

Open Science in Action: Preregistration

IDEA

Releasing your research question and study design BEFORE beginning your project

Benefits:

- Allows for feedback at the beginning of your project
- Eliminates bias
- Ensures reproducibility of results
- Lets people know research is being done on this topic

Open Science in Action: Preregistration

IDEA

Example Preregistration Template:

- Research Question
- Study Description/Justification
- Study Design
- Data Collection Procedures
- Variables being studied (e.g., frequency of caffeine consumption)

Open Science in Action: Pre-registration

Surveying the landscape of CIHR-funded research data sharing practices: An analysis of the published literature

Public registration

- Overview
- Files
- Wiki
- Components 0
- Links 0
- Analytics
- Comments 0

Summary

Provide a narrative summary of what is contained in this registration or how it differs from prior registrations. If this project contains documents for a preregistration, please note that here.

INTRODUCTION: This study will aim to accomplish two specific goals by assessing the availability of health sciences research datasets funded by the Canadian Institutes of Health Research (CIHR). The first goal will be to understand the Canadian data sharing landscape by reviewing how and where Canadian health sciences researchers share their data. The second goal will be to compare Canadian researchers' current data sharing practices to the Tri-agency's proposed framework for research data management and sharing. The information gathered from this study will be used to identify gaps within the Canadian data sharing landscape, and help inform the future development of data policy, infrastructure and research data management support by highlighting the key challenges and opportunities with respect to data sharing in a Canadian context.

METHODS: This study will identify all CIHR-funded articles that have indicated whether or not they have shared the research data underlying their published results using PubMed and PubMed Central's (PMC) dataset search filters. Using PubMed Central, this study will identify CIHR-funded articles that include a data availability statement or include data citations. An additional set of articles will be identified in PubMed using the data filter, which locates articles that directly link to National Institutes of Health-specific or external data repositories. These respective searches will be combined with CIHR-related terms, including both its English and French pronunciation, in the grants information field of both databases. It is anticipated that this search will retrieve close to 5000 articles across both databases.

Contributors

Kevin Read

Description

This study will aim to accomplish two specific goals by assessing the availability of health sciences research datasets funded by the Canadian Institutes of Health Research (CIHR). The first goal will be to understand the Canadian data sharing landscape by reviewing how and where Canadian health sciences researchers share their data. The second goal will be to

Show more

Registration type

Open-Ended Registration

Date registered

January 17, 2020

Date created

January 17, 2020

Registered from

osf.io/n9jv5

https://osf.io/wjgdb

University of Saskatchewan

Checkpoint 1: Uploading your research question

Publicly share your research question and justification before starting your research

Consider:

- Communicating your research question and justification in plain language
- Will be a viewers first exposure to your research
- Always include your names so that you can be credited

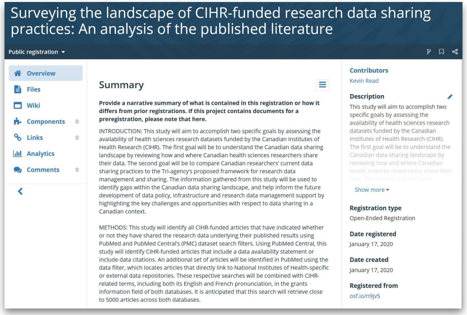

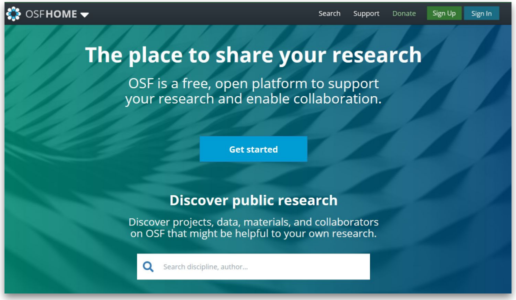

Open Science **in Action**

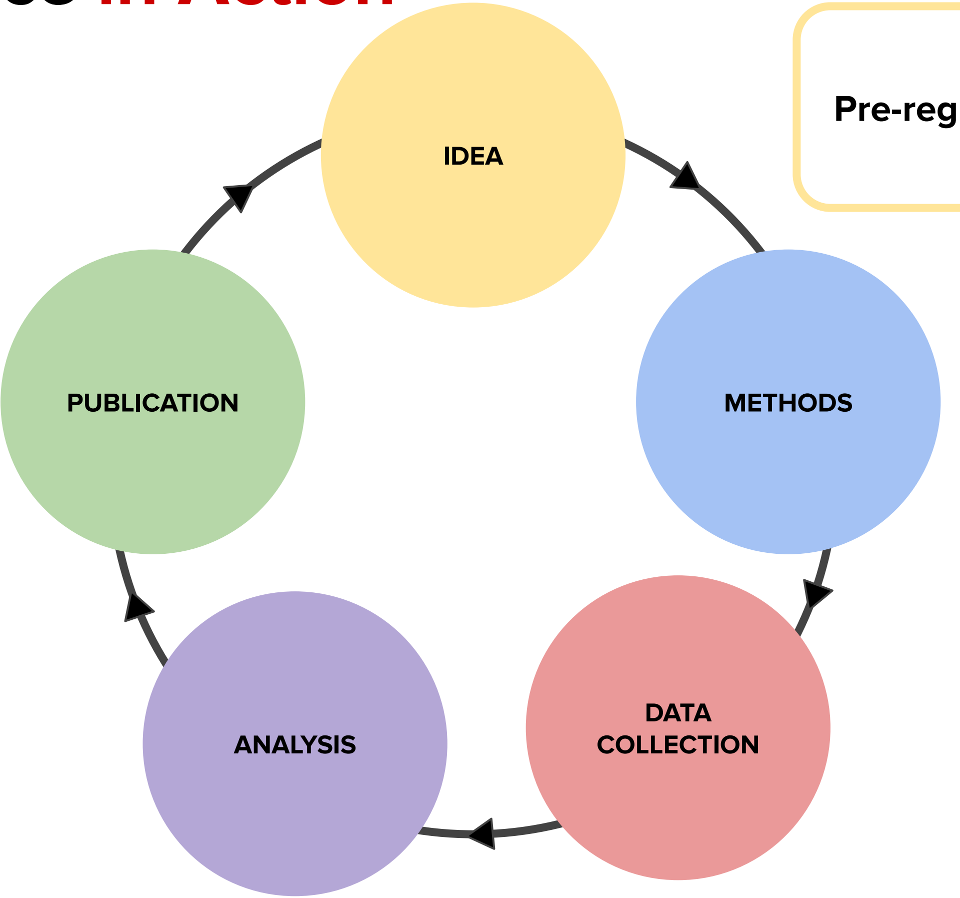

Pre-register your project

Share your methods, instruments, etc. when possible

Open Science in Action: Sharing Methods and Tools

METHODS

Sharing the instruments, tools, etc. that you create will allow people to see **HOW** you are conducting your research

Benefits:

- Eliminates bias
- Allows others to potentially use your instruments/tools for their own research
- Opportunity to gain feedback from peers
- Provides an opportunity for you to make your research understandable to others

UNIVERSITY OF SASKATCHEWAN

Open Science in Action: Sharing Methods and Tools

METHODS

Example:

- Describing an experiment
- Sharing a questionnaire
- Sharing a survey
- Sharing the software you will use to collect data

Open Science in Action

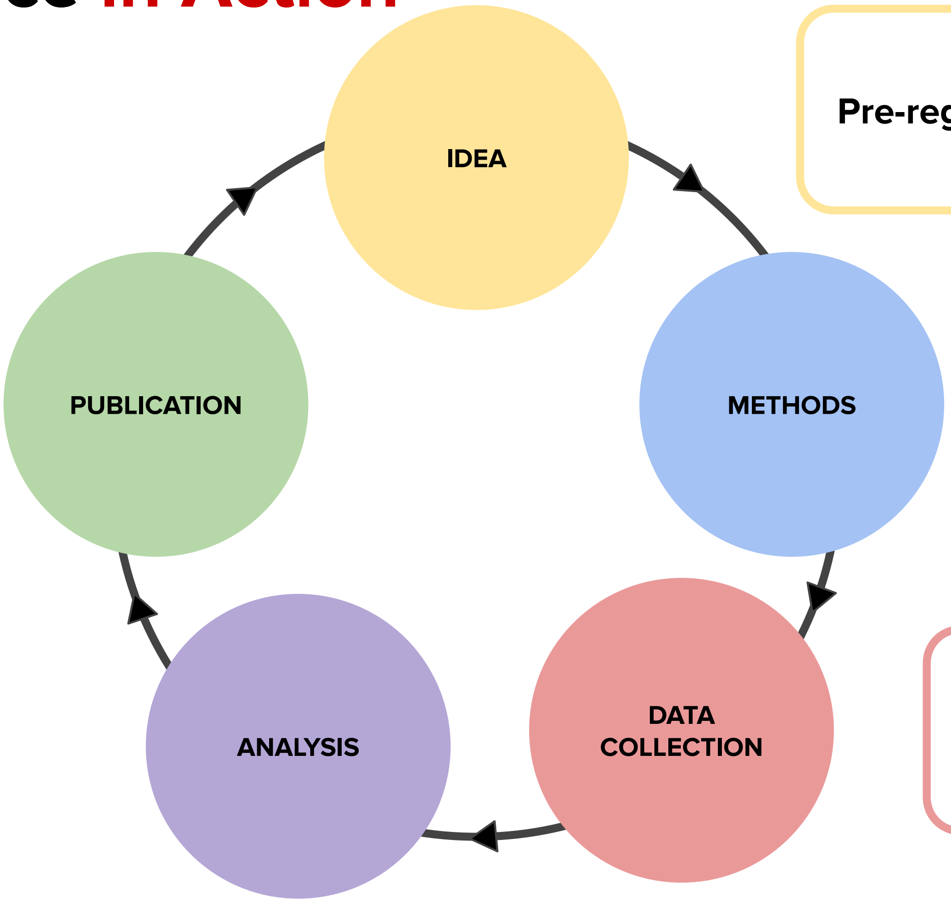

Pre-register your project

Share your methods, instruments, etc. when possible

Describe your data so it can be understood by others

University of Saskatchewan

Open Science in Action: Make Data Understandable

DATA COLLECTION

Make sure everyone understands and can interpret the data that you collect.

Benefits:

- Your data can be understood by others
- Your data can be *reused* by others
- Your data provides a bigger picture of your research project

Is Your Data Understandable?

??????

p1	p2	p3	p4	p5
1	0	3	Active	Strong
1	0	3	Inactive	Moderate
4	0	2	Unknown	Unknown
3	1	5	Inactive	Weak
0	1	2	Inactive	Weak

UNIVERSITY OF SASKATCHEWAN

Open Science in Action: Make Data Understandable

DATA COLLECTION

Example:

Create a data dictionary of your data

Maricopa Regional Household Activity Survey: 2001
Matrix of Data Items

NuStats
Revised 4/10/01
Pg 1 of 12

Item	Var Name	Variable Description	Data Type	Width	Values
H-1	SAMPN	HH ID Number	N	7	
H-2	HHADDR	Household Location Reference Number	N	10	
H-3	AREATYPE	Area Type	N	1	1=CBD, 2=Outlying CBD, 3=Mixed Urban, 4=Suburban, 5=Rural, 9=Apache Junction
H-4	PHONE	HH Phone number	C	10	
H-5	LANG	Interview Language	N	1	1=English, 2=Spanish
H-6	HHVEH	Number of motorized vehicles available for use by HH members	N	2	Ordinal Variable; 98=Don't know; 99=Refused
H-7	BIKES	Number of bicycles	N	2	Ordinal Variable; 98=Don't know; 99=Refused
H-8	HHSIZE	No. of persons in household	N	2	
H-9	ETHN	HH Ethnicity	N	1	ETHN
H-10	O_ETHN	Other Ethnicity	C	30	
H-11	WRKR	Number of HH Workers	N	2	
H-12	DWELTYPE	Type of dwelling unit	N	1	DWELTYPE
H-13	DWELO	Other type of dwelling	C	30	
H-14	OWN	Owner/Renter Status	N	1	1=Own/buying; 2=Rent; 7=Other, 8=DK, 9=RF
H-15	O_OWN	Other Owner/Renter Status	C	30	
H-16	TENURE	Tenure at dwelling unit	N	1	1=<1 yr, 2=1-3 yrs, 3=4-5 yrs, 4=more than 5 yrs, 9=DK/RF
H-17	COMPTR	Computer Ownership Status	N	1	1=Yes; 2=No; 8=DK; 9=RF
H-18	INTERNET	Internet Service Status	N	1	1=Yes; 2=No; 8=DK; 9=RF
H-19	INTACC	Internet Access Type	N	1	0=NONE, 1=Dial-up/Modem with tones, 2=High speed connection, 3=Web TV, 7=Other, 9=DK/RF
H-20	INTACCO	Other Access Type	C	30	
H-21	PHLNS	Number of telephone lines	N	1	Ordinal Variable, 98=DK, 99=RF
H-22	FAXMODEM	Number of telephone lines used exclusively for Fax or Modem	N	1	Ordinal Variable, 98=DK, 99=RF
H-24	NOPHN	Lack of phone service	N	1	1=Yes; 2=No; 8=DK; 9=RF
H-25	LENGTH	Lack of phone service	N	1	LENGTH
H-26	INCAT	Income Category	N	1	1=Below 35K, 2=Above 35K, 8=DK, 9=RF
H-27	INCOME	Total 2000 annual household income	N	2	INCOME

j:\projects\maricopa\data\bc2bd132-9b61-404b-936d-8331c24fe35d.xls

https://shatterlion.info/wp-content/uploads/2018/01/data-dictionary-template-data-dictionary-template-dfxkkz16.jpg

Checkpoint 2: Sharing Survey Questions & Building a Data Dictionary

Survey Q's

4. What is your current position at the University of Saskatchewan?

- ◯ Undergraduate student (Canadian student)
- ◯ Undergraduate student (International student)
- ◯ Graduate student (Canadian student)
- ◯ Graduate student (International student)
- ◯ Post-doctoral fellow
- ◯ Faculty/staff member
- ◯ Other (please list)

Raw data

	J	K	L	M	N	O	P	Q	R	S	T	U	V	W	X	Y	Z	AA
1	I consent	What is your age in years?	What is your	What is your current positic		What best de	Do you cu	Have you	Do you ha	Do you cu	What forms of social media do you currently use? (Choose all that apply)							
2	Respons	Open-Ended Response	Response	Response	Other (please	Response	Respons	Respons	Respons	Respons	Facebook	Twitter	Instagram	Reddit	Pinterest	YouTube	LinkedIn	Blogs (e.g
3	1	19	1	1		1	1	1	1	2	1		3	4	5	6		
4	1																	
5	1	24	1	1		2	1	2	1	2	1		3		5	6		
6	1	27	2	3		1	1	1	1	2	1	2	3	4		6	7	
7	1	20	1	1		2	1	2	1	2					5	6		
8	1	19	1	1		1	1	1	1	2	1		3		5	6		
9	1	40	1	6		1	1	1	1	2	1	2	3		5	6	7	
10	1	20	1	1		1	1	2	1	2	1	2	3			6		
11	1	21	1	1		1	1	2	1	2	1		3		5			
12	1	39	1	6		1	2	1	1	2	1					6		
13	1	27	1	6		1	1	2	2	2	1	2	3	4		6		
14	1	38	1	1		1	1	2	1	2	1				5	6		
15	1	18	1	1		1	1	1	3	2	1	2	3	4	5	6		
16	1	32	1	1		3	1	1	2	2	1		3		5			
17	1	21	2	1		1	1	1	1	2	1	2	3	4		6	7	
18	1	21	1	1		1	1	1	1	2		2		4		6		
19	1	18	1	1		2	1	1	1	2	1		3	4	5			
20	1	21	2	1		1	1	1	2	2	1		3	4		6		
21	1	25	1	1		1	1	2	1	2	1		3	4				
22	1	26	2	4		2	1	1	1	2	1	2		4		6	7	
23	1	30	1	1		2	1	1	2	2	1	2	3	4	5	6		
24	1	20	1	1		3	1	1	1	2			3	4		6		
25	1	20	1	1		1	1	2	1	2	1		3		5	6		
26	1	21	1	1		1	1	1	1	2	1		3		5	6	7	
27	1	28	1	6		1	1	2	1	2	1					6		
28	1	50	1	0	auditing stude	1	1	1	2	1								
29	1	23	1	1		1	1	1	1	2	1		3		5		7	
30	1	19	1			1	1	1	1	2	1		3		5	6		
31	1	23	1	1		1	1	1	2	2	1		3		5	6		
32	1	37	1	6		1	1	1	1	2	1		3		5	6	7	
33	1	23	1	1		1	1	2	1	2	1				5	6		
34	1	20	1	1		1	1	1	1	2	1	2	3		5	6		
35	1	19	1	1		1	1	1	2	2			3			6		
36	1	21	1	1		1	1	2	1	2	1	2	3	4		6	7	
37	1	63	1	6		1	1	2	1	2		2				6	7	
38	1	28	2	1		1	2	1	1	2	1		3	4		6	7	
39	1	19	1	1		1	1	1	1	2			3					
40	1	25	3	1		2	1	1	2	2	1	2	3			6		
41	1	28	1	1		1	2	1	1	2	1		3	4		6		

University of Saskatchewan

Checkpoint 2: Building a Data Dictionary

Template (Available in the Open Science Module on Canvas):

	A	B	C	D	E
1	**Variable Name**	**Variable Definition**	**Variable Type**	**Variable Values**	**Variable Instructions (if necessary)**
2	age_years	The age of participants in years	text	N/A	Must enter a number value for age
3	Do you take iron supplements?	Assessment of participant's iron intake	multiple choice	0 - No; 1 - Yes; 2 - Don't Know	N/A
4	What are your three favourite foods?	Participant's top three selections of their favourite foods	checkbox	1 - Chocolate; 2 - Pizza; 3 - Steak Dinner; 4 - Sour Patch Kids; 5 - other	Other is a free text field and can be entered in manually by participants
5					
6					
7					
8					
9					

UNIVERSITY OF SASKATCHEWAN

Checkpoint 2: Building a Data Dictionary

	A	B	C	D	E
1	**Variable Name**	**Variable Definition**	**Variable Type**	**Variable Values**	**Variable Instructions (if necessary)**
2	age_years	The age of participants in years	text	N/A	Must enter a number value for age
3	Do you take iron supplements?	Assessment of participant's iron intake	multiple choice	0 - No; 1 - Yes; 2 - Don't Know	N/A
4	What are your three favourite foods?	Participant's top three selections of their favourite foods	checkbox	1 - Chocolate; 2 - Pizza; 3 - Steak Dinner; 4 - Sour Patch Kids; 5 - other	Other is a free text field and can be entered in manually by participants
5					
6					
7					
8					
9					

Checkpoint 2: Building a Data Dictionary

FOCUS ON UNDERSTANDABILITY/TRANSPARENCY!

	A	B	C	D	E
1	**Variable Name**	**Variable Definition**	**Variable Type**	**Variable Values**	**Variable Instructions (if necessary)**
2	age_years	The age of participants in years	text	N/A	Must enter a number value for age
3	Do you take iron supplements?	Assessment of participant's iron intake	multiple choice	0 - No; 1 - Yes; 2 - Don't Know	N/A
4	What are your three favourite foods?	Participant's top three selections of their favourite foods	checkbox	1 - Chocolate; 2 - Pizza; 3 - Steak Dinner; 4 - Sour Patch Kids; 5 - other	Other is a free text field and can be entered in manually by participants
5					
6					
7					
8					
9					

Open Science in Action

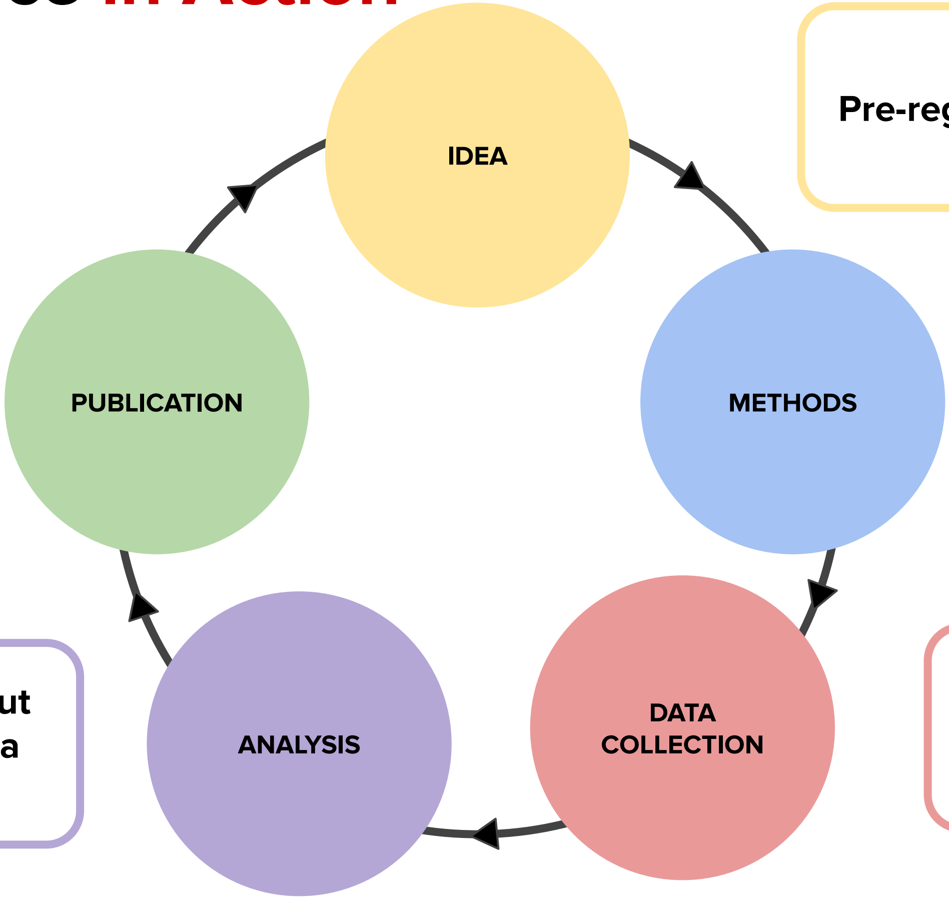

Pre-register your project

Share your methods, instruments, etc. when possible

Describe your data so it can be understood by others

Be transparent about and share your data analysis plan

Open Science in Action: Build an analysis plan

ANALYSIS

Provide a description of how you will explore your data after it has been collected.

Benefits:

- Holds you accountable to how you will analyze your data
- Eliminates the risk of manipulation of data
- Provides clear instructions to a user about how you transformed your data

Open Science in Action: Build an analysis plan

ANALYSIS

Examples:

- "I created pivot tables using the height and weight variables"
- "We averaged the caffeine consumption of adults between the ages of 20 and 25"
- "We examined how frequently male and female students eat breakfast by looking at the **gender** and **student_bkfst** variables"

What is included in a data analysis plan?

A summary of what the document is about

A description of every data analysis procedure you complete, including:

- The type of analysis (e.g, average, mean, count)
- The variables you analyzed and the value your are exploring (e.g., gender = female, coffee consumption = 5 times a day)
- Any figures or summary results that you found

Reference/link to your survey and/or data dictionary for more context

Example: My own research

Analysis 1: Counting Data Sharing Methods

Using the data collection instrument: *CIHR-funded Data Sharing Instrument*, we counted (using COUNT= function in Microsoft Excel) the methods of data sharing.

Variables used:

- Is data available? = 1
- Is data accessible? = 1
- Was data sharing not possible? = 1
- Was there no evidence of data sharing? = 1

Generated figure:

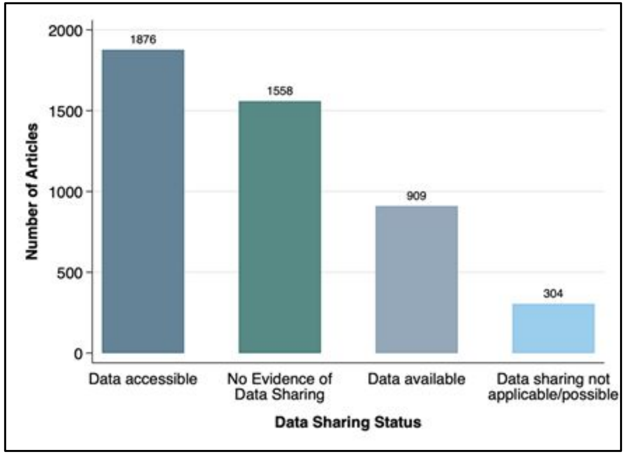

Related documents:

- CIHR-funded Data Sharing Instrument
- Data Dictionary

Example: My own research continued...

Analysis 1: Counting Data Sharing Methods

Using the data collection instrument titled: CIHR-funded Data Sharing Instrument, we counted the different methods of data sharing.

Variables used:

- Is data available? = 1
- Is data accessible? = 1
- Was data sharing not possible? = 1
- Was there no evidence of data sharing? = 1

Related documents:

- CIHR-funded Data Sharing Instrument
- Data Dictionary

Analysis 2: Averaging CIHR-funded Data Accessibility

We averaged the total number of times a researcher with CIHR-funded made their research data accessible to the public.

Variables used:

- Grant agency = 1 and Is data accessible? = 1

Related documents:

- CIHR-funded Data Sharing Instrument
- Data Dictionary

UNIVERSITY OF SASKATCHEWAN

Example: My own research continued...

Analysis 1: Counting Data Sharing Methods ← **Title**

Using the data collection instrument titled: CIHR-funded Data Sharing Instrument, we counted the different methods of data sharing. ← **Description**

Variables used: ← **Variables w/ values**

- Is data available? = 1
- Is data accessible? = 1
- Was data sharing not possible? = 1
- Was there no evidence of data sharing? = 1

Related documents: ← **Related documents**

- CIHR-funded Data Sharing Instrument
- Data Dictionary

Analysis 2: Averaging CIHR-funded Data Accessibility ← **Repeat for each new analysis**

We averaged the total number of times a researcher with CIHR-funded made their research data accessible to the public.

Variables used:

- Grant agency = 1 and Is data accessible? = 1

Related documents:

- CIHR-funded Data Sharing Instrument
- Data Dictionary

Open Science in Action

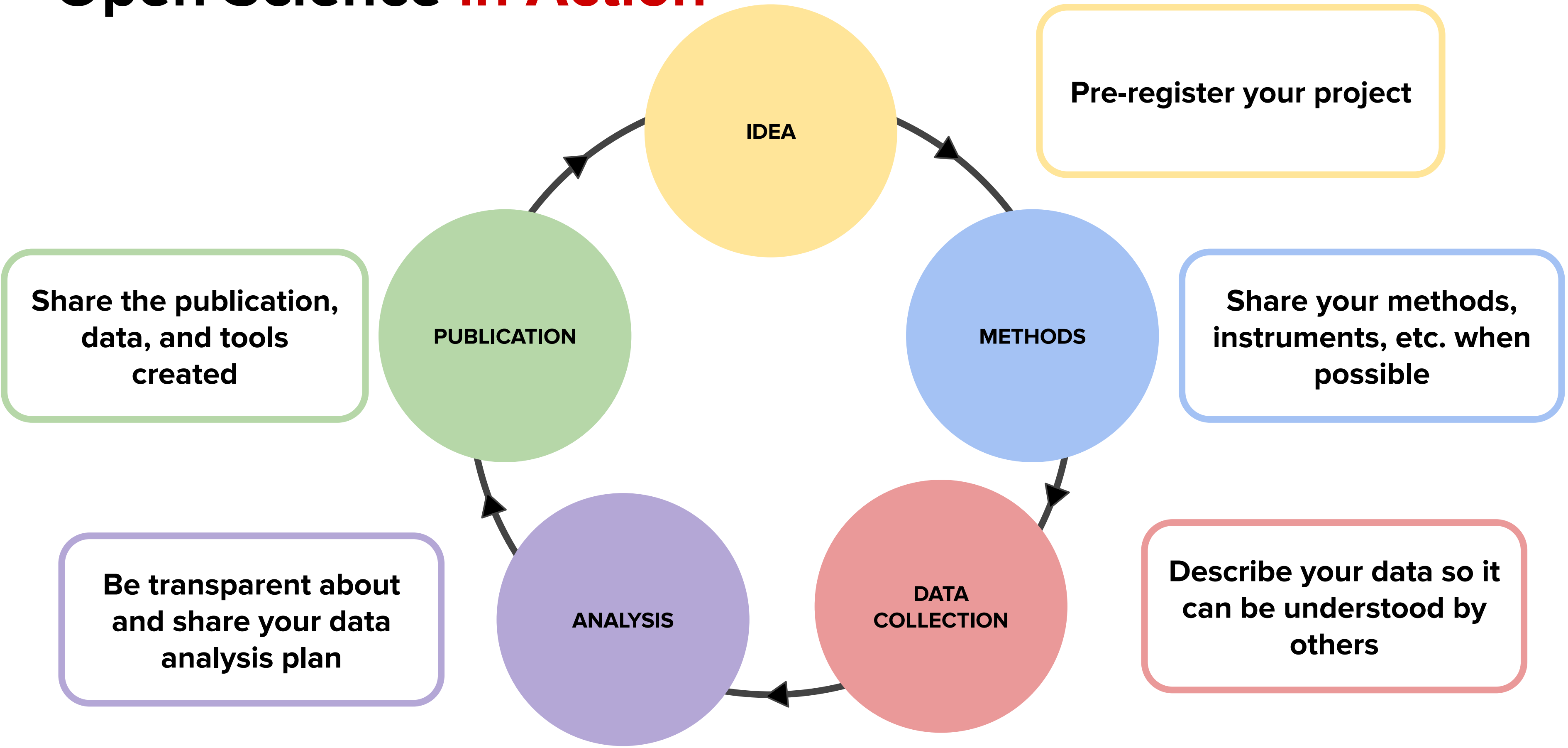

Open Science in Action: Share your research

PUBLICATION

Share all products of research including your final paper, data (when possible), conference presentation, poster, software, etc.

Benefits:

- Others can see the full breadth of your research
- Your research will be reproducible
- You research will accessible to everyone

Open Science in Action: Share your research

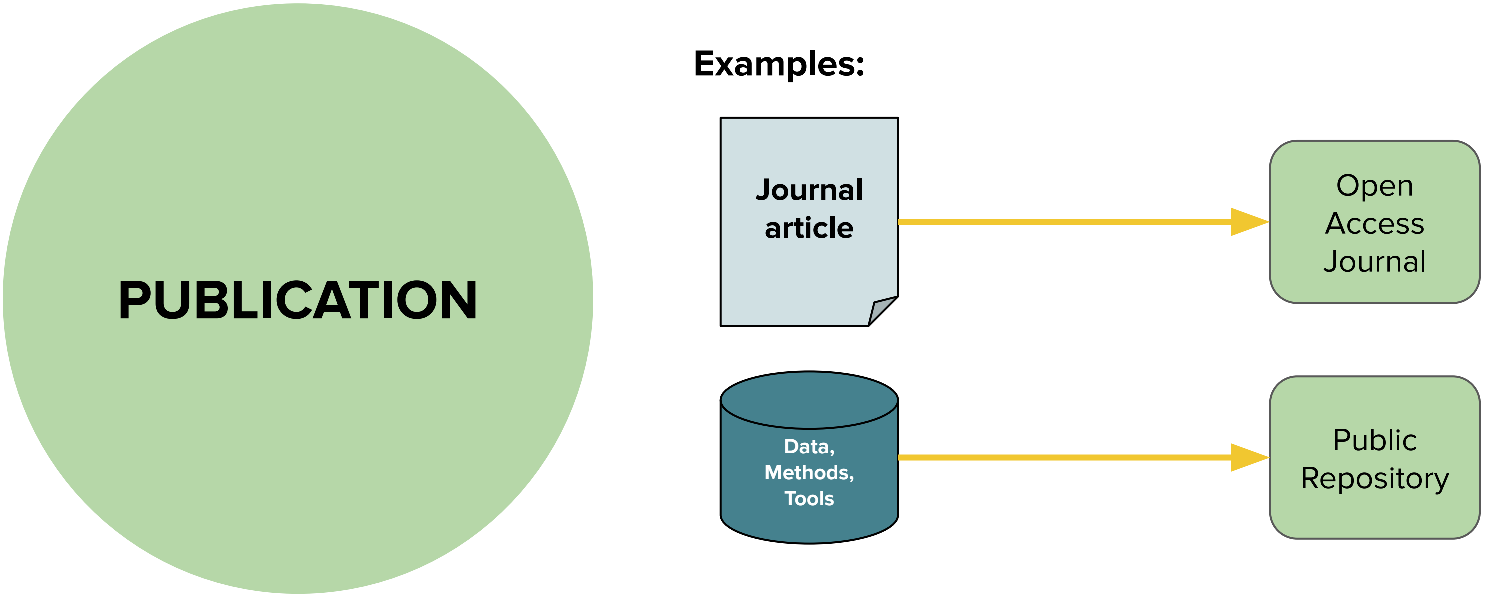

Open Science Framework (OSF)

Open Science Framework

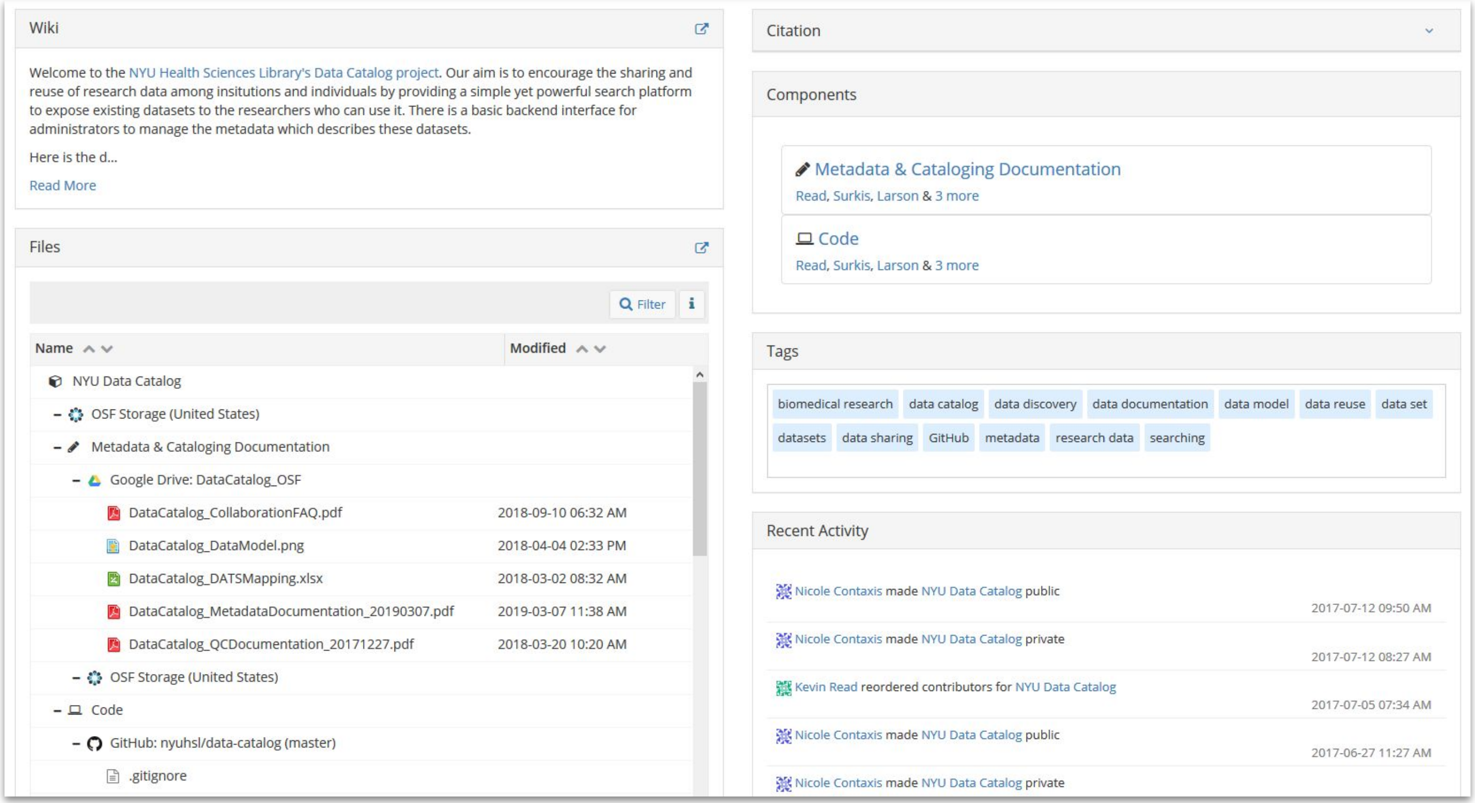

https://osf.io/

Exercise: Create an OSF Account

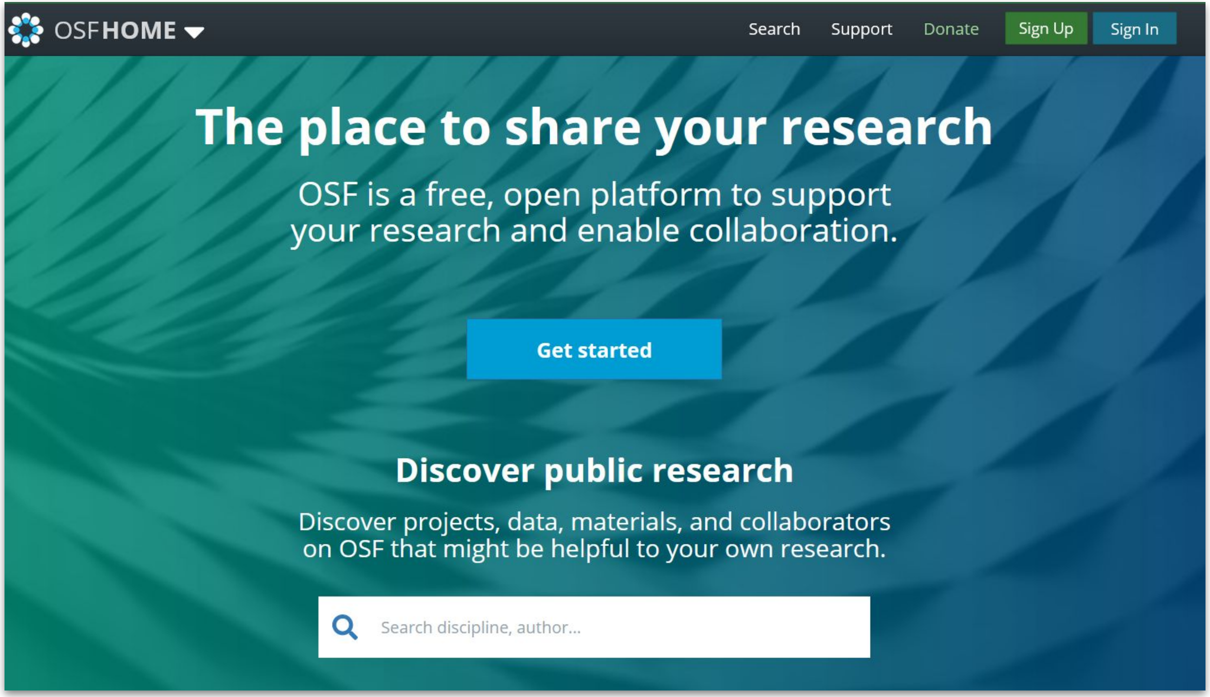

https://osf.io/

Open Science Framework Panopto Videos

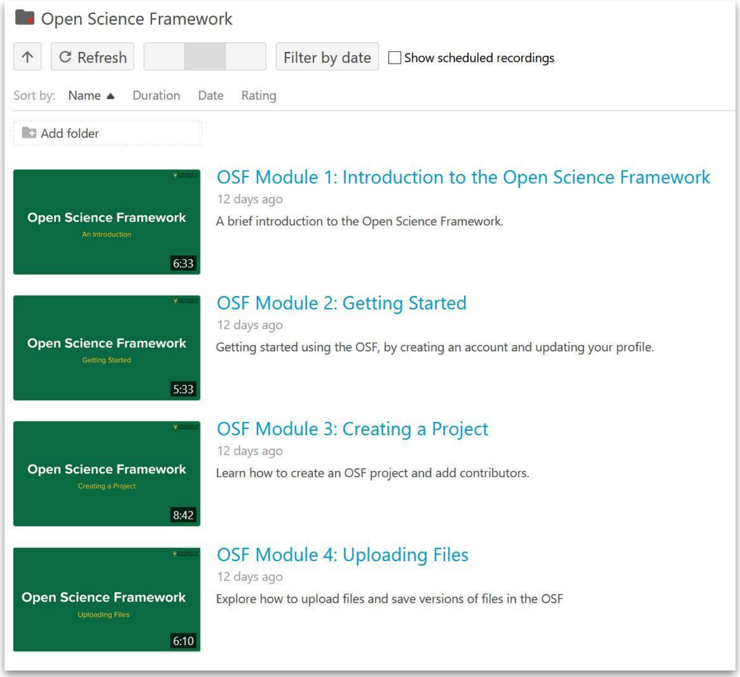

https://usask.cloud.panopto.eu/Panopto/Pages/Sessions/List.aspx?folderID=655bd58f-9927-4ad0-a414-ac2a0106df48

Open Science in Action: My Own Research

Preregistration

Research Products Available Online

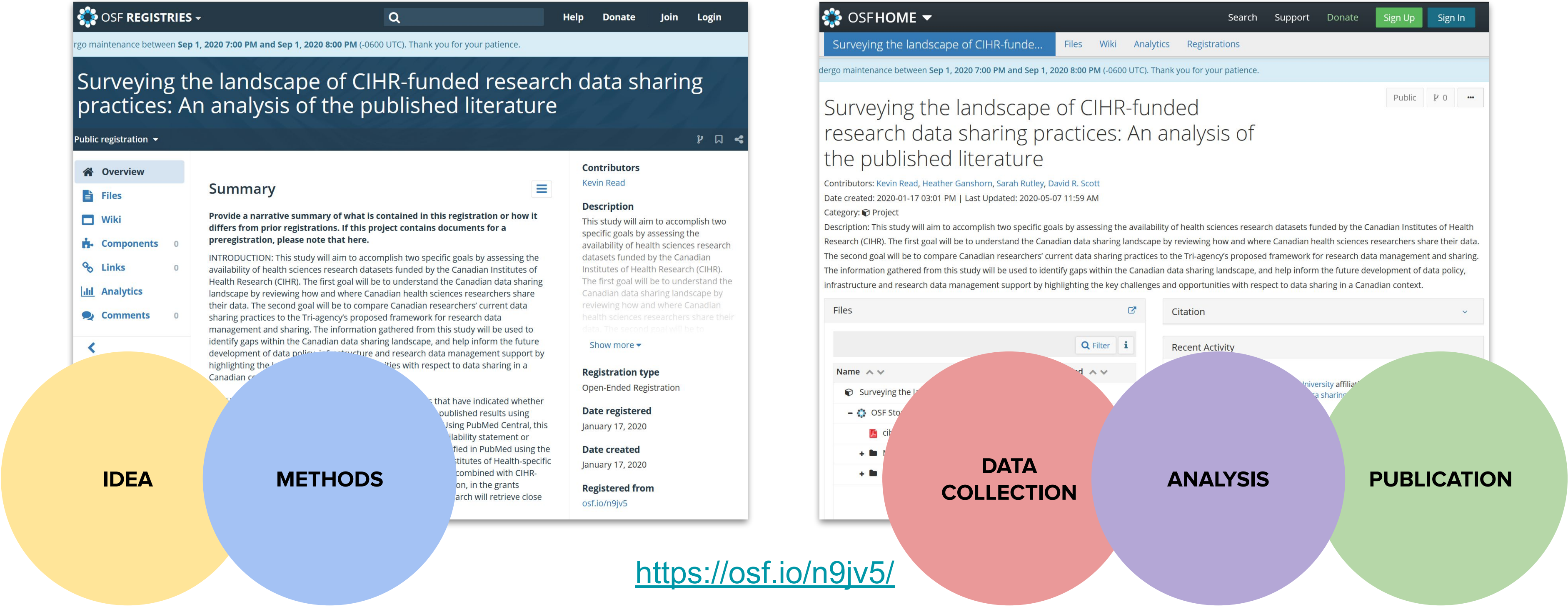

https://osf.io/n9jv5/

Open Science in Action: Getting Credit

Research Products Available Online

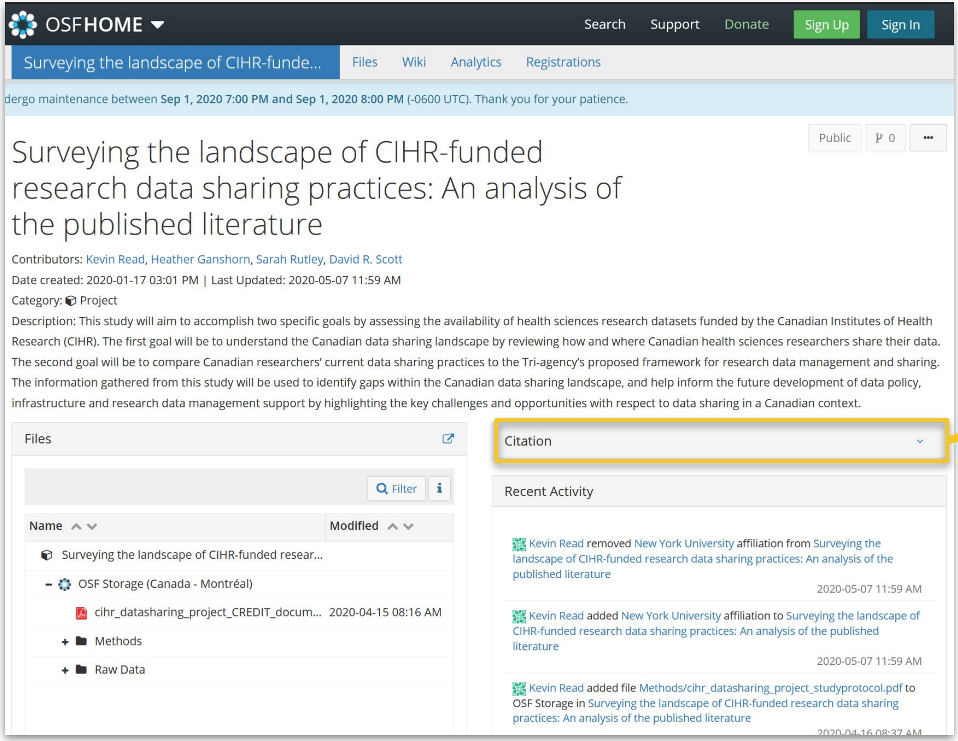

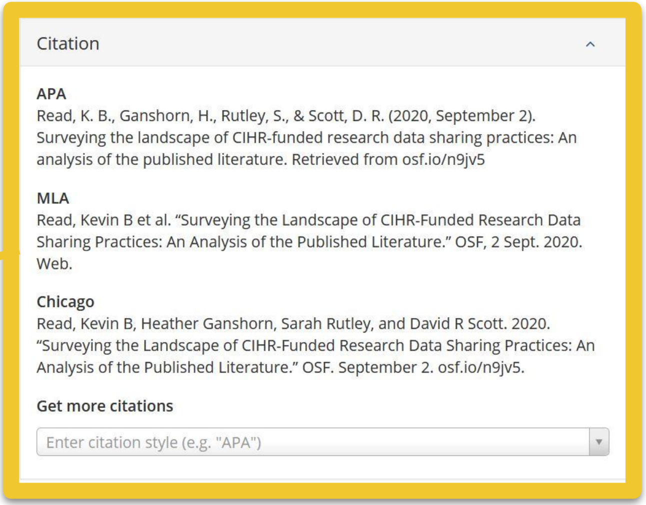

https://osf.io/n9jv5/

Open Science: Transparent process

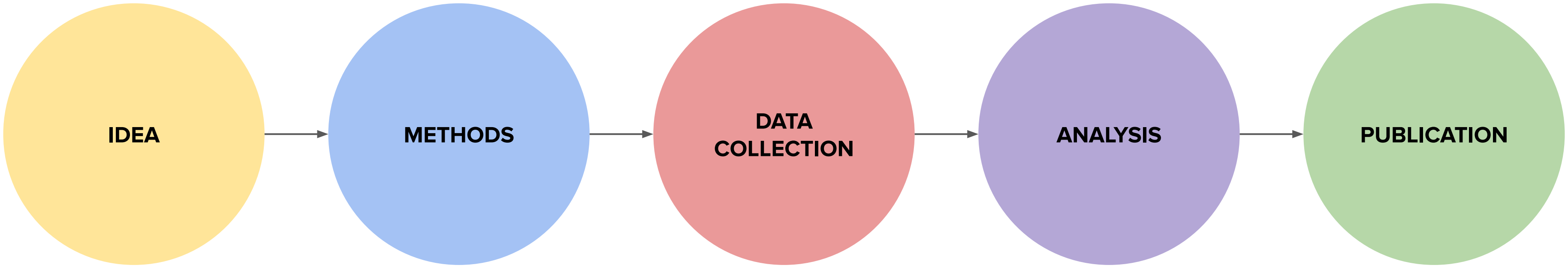

Open Science: Transparent process

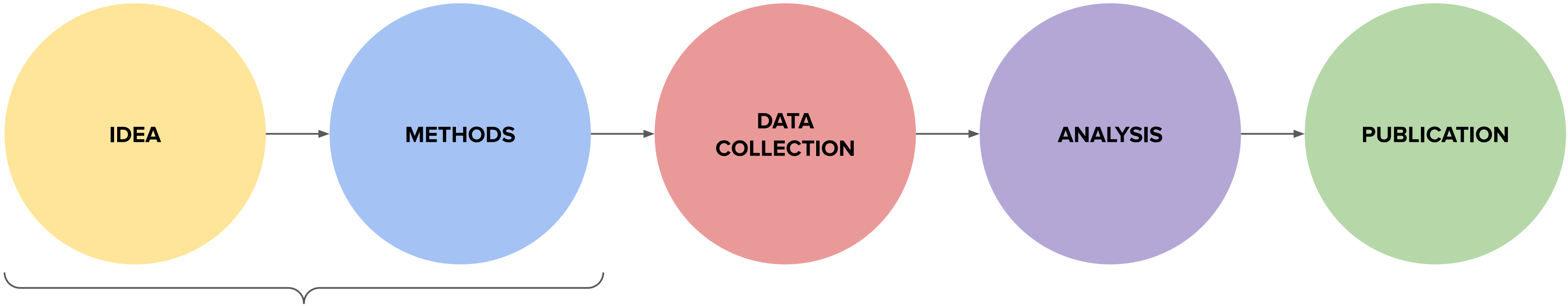

Preregister study
Share methods, instruments, tools

Open Science: Transparent process

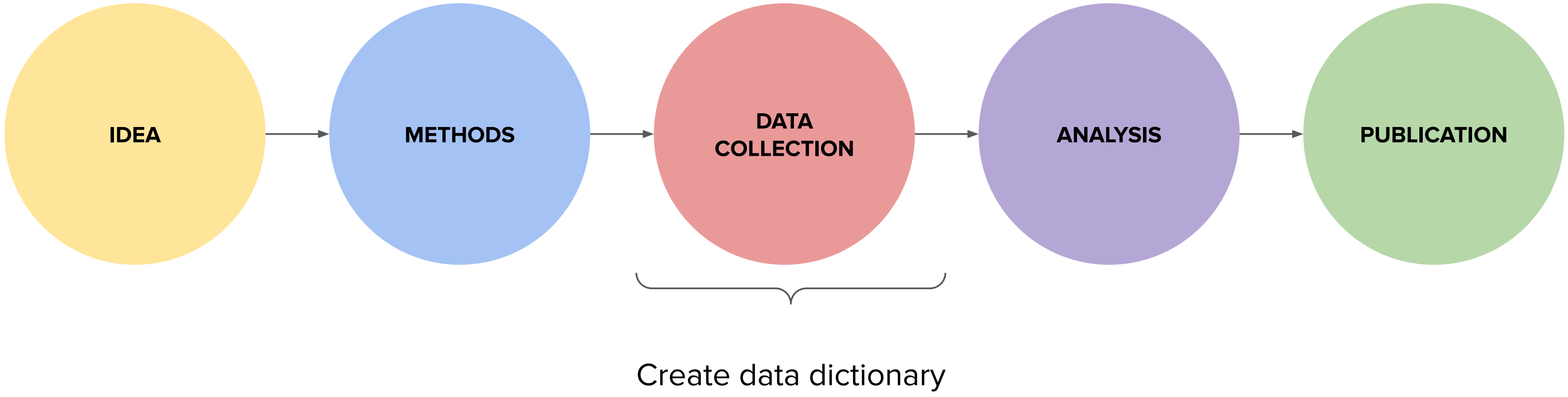

Open Science: Transparent process

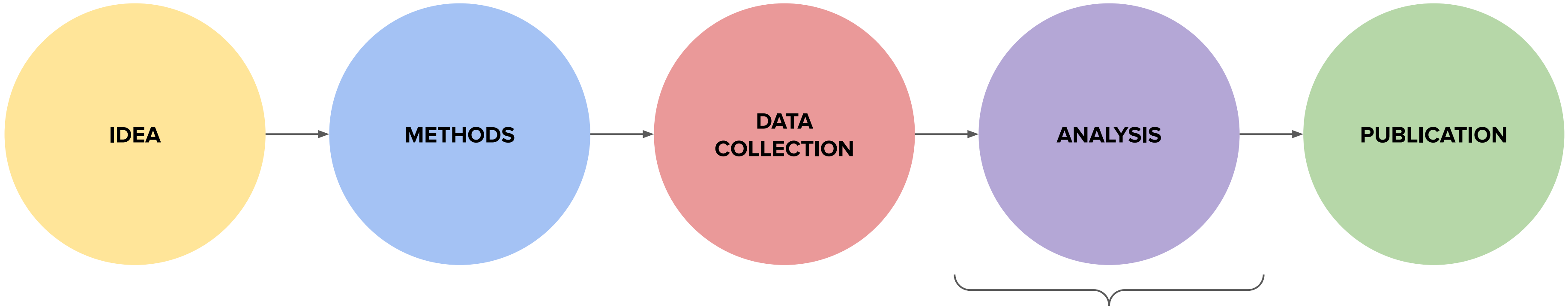

Describe data analysis
Share data analysis plan

UNIVERSITY OF SASKATCHEWAN

Open Science: Transparent process

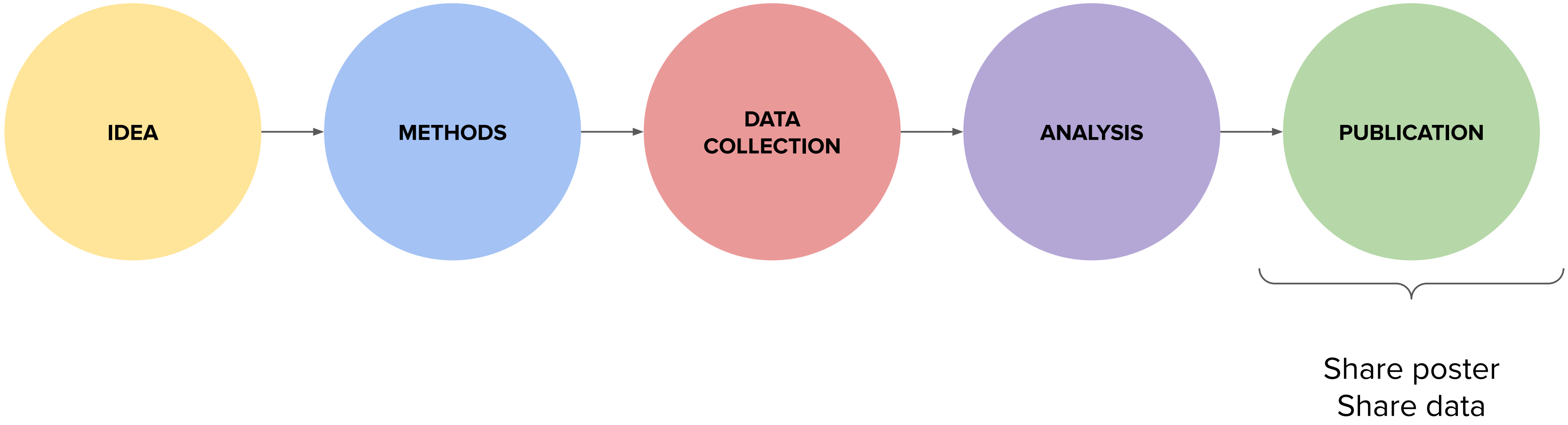

University of Saskatchewan

Open Science: Transparent ONGOING process

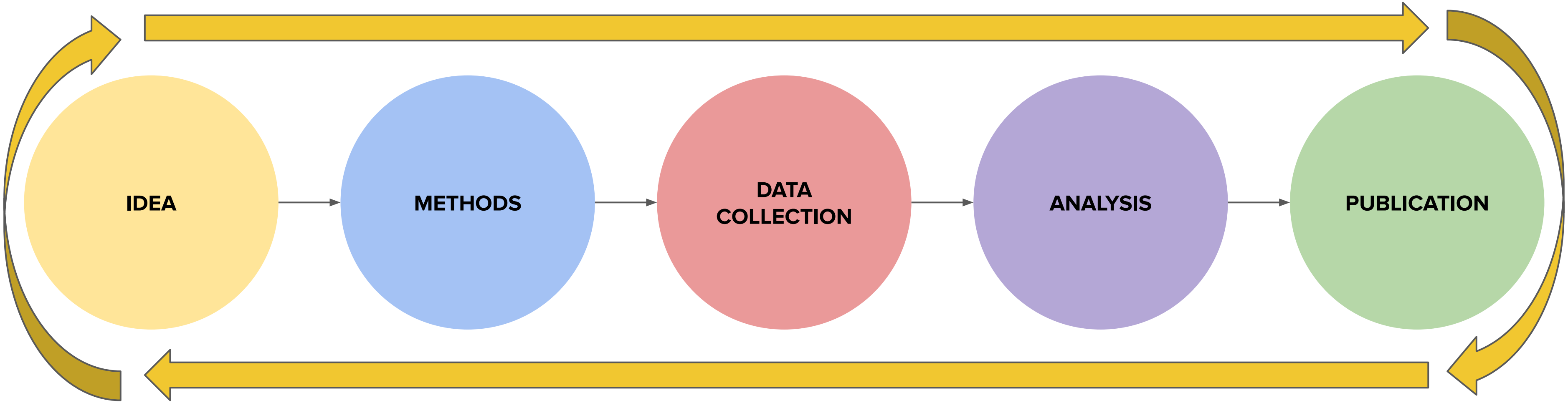

Open Science: Provides access to new material

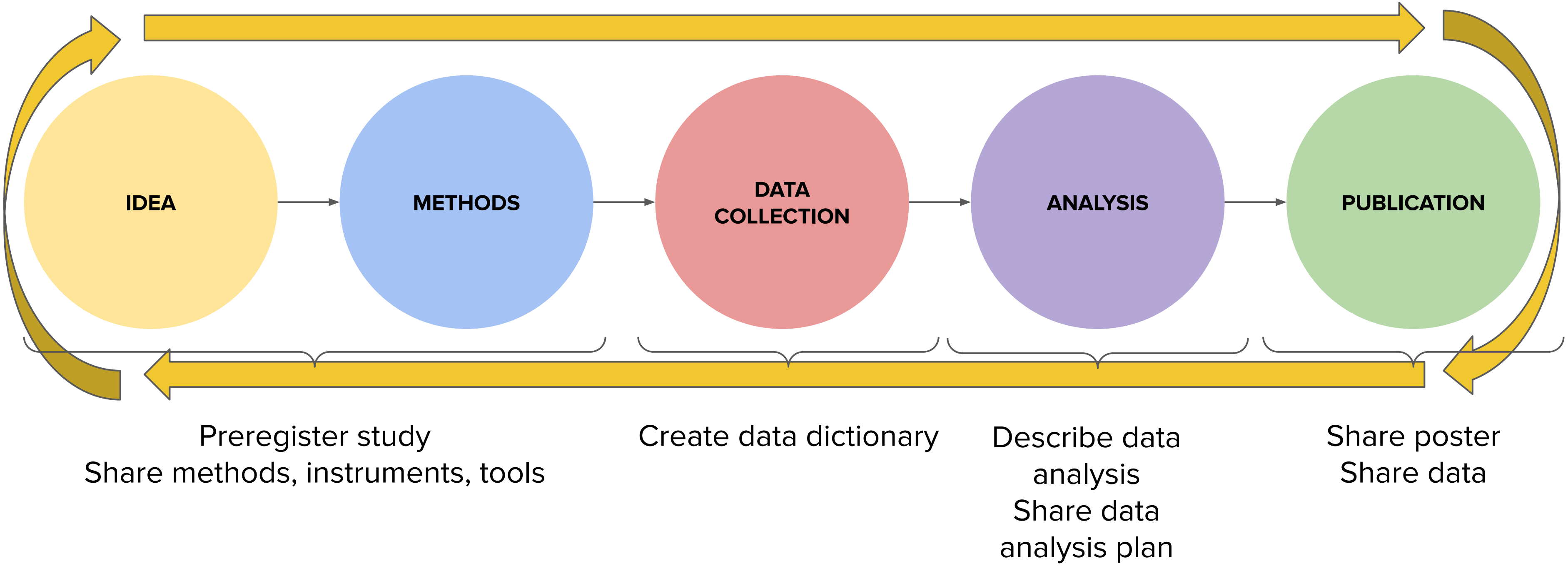

UNIVERSITY OF SASKATCHEWAN

Open Science in NUTR 230

University of Saskatchewan

Open Science Checkpoints

Checkpoint	Open Science Component
Checkpoint 1: Submission of Research Question and Justification	Students create a collaborative **OSF** project and upload the finalized research question and justification. Share the link on the project pages of Canvas. **Due: Sept 21**
Checkpoint 2: Submission of Survey Questions	Student create a data dictionary of their survey questions and upload to their **OSF** project. **Due: Oct 7**
Checkpoint 3: Submission of Draft Introduction & Methods Section of Poster	Students upload their introduction and methods to their **OSF** project. **Due: Nov 5**
Checkpoint 4: Data Analysis Progress and Draft Poster	Students write a summary of the analysis procedures they have applied to the data and upload it to their **OSF** project. **Due: Nov 25**
Final product: Completed poster	Students upload their completed poster to their **OSF** project and cite their OSF project in their poster. **Due: Nov 30**

Open Science Checkpoints: File Naming

Before uploading documents to the Open Science Framework, use this file naming convention for clarity:

projectname_documenttype_YYYYMMDD[date]

Examples:

studentexerciselevels_datadictionary_20200901

caffeineconsumption_researchquestion_20201029

plantbaseddiets_introandmethods_20200812

Summary

Consider the value of making your research openly available

Continually ask yourself whether your project would be understandable to someone unfamiliar with you work

Complete the checkpoints with the idea that your research could be viewed by anyone

Open science practices can improve the quality and transparency of research for the better!

Resources

FYRE-specific Open Science Framework Training Videos: https://usask.cloud.panopto.eu/Panopto/Pages/Sessions/List.aspx?folderID=655bd58f-9927-4ad0-a414-ac2a0106df48

How to Make a Data Dictionary: https://help.osf.io/hc/en-us/articles/360019739054-How-to-Make-a-Data-Dictionary

What is Open Science? https://www.fosteropenscience.eu/node/2269

Comprehensive Open Science Framework Guides: https://help.osf.io/hc/en-us

Canvas Modules

- First Year Research Experience (FYRE) Resources and Activities
  - Open Science Resources
    - Open Science Framework Team Projects Page
    - Open Science Lecture Slides
    - Open Science Framework Tutorials
    - Open Science File Naming Guidance
    - Data Dictionary Template
      - How to Make a Data Dictionary Guide
    - Data Analysis Plan Template

University of Saskatchewan

References

Allen C, Mehler DMA (2019) Open science challenges, benefits and tips in early career and beyond. PLOS Biology 17(12): e3000587. https://doi.org/10.1371/journal.pbio.3000587

Goodman, S. N., Fanelli, D., & Ioannidis, J. P. A. (2016). What does research reproducibility mean? *Science Translational Medicine*, *8*(341), 341ps12 LP-341ps12. https://doi.org/10.1126/scitranslmed.aaf5027

Society, T. R. (2012). *Science as an open enterprise* (Issue June). The Royal Society. https://royalsociety.org/topics-policy/projects/science-public-enterprise/report/

UNIVERSITY OF SASKATCHEWAN

Questions?

kevin.read@usask.ca

(or ask in the Canvas FYRE Lounge)
