# Supplementary material for: Integrating open science education into an undergraduate health professional research program
Source: J Med Libr Assoc. 2022 Oct 1;110(4):429–37. doi: 10.5195/jmla.2022.1457 (PMC10124608; doi:10.5195/jmla.2022.1457)
Supplement: Supplementary file 3 — Appendix C: Course Instructions [file jmla-110-4-429-s03.pdf]

NUTR 230
Professional Practice I
FYRE Project
Value: 30% of final grade

Research is an important part of the work of dietitians in all areas of practice. Research in numerous areas (e.g., management, basic sciences, food science, behavioural and social sciences, nutritional science, dietetics) has formed the foundation of the work of dietitians. In addition, new emerging research conducted by dietitians and others also helps improve the practice of dietitians and ultimately the nutritional health and wellbeing of the overall population.

As part of NUTR 230, you will work in pre-assigned teams of 3-4 students to conduct a research project to investigate the practices, knowledge and/or attitudes of the University of Saskatchewan community on a nutrition, food, and/or dietetic related topic of your choice. This project will help you to begin to understand the process of conducting research which is an important skill for dietitians in all areas of practice.

As part of this First Year Research Experience (FYRE) project, you will be asked to **develop a research question** that you wish to investigate. You will then **investigate your question** by having members of the University of Saskatchewan community complete three to five survey questions (mounted on SurveyMonkey) to answer your question. You will then analyze your data and create a research poster with your findings. You will submit your research poster for evaluation and **share your results with your classmates, as well as others from the College of Pharmacy and Nutrition and the University of Saskatchewan campus community and beyond in a virtual poster showcase during the last week of Fall 2020 classes**.

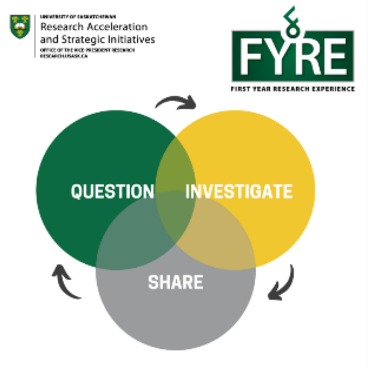

Students will also be introduced to best practices and tools of Open Science (OS) as part of the NUTR 230 FYRE project. OS has been defined as the practice of making everything in the research process fully and openly available, creating transparency, and driving discovery by allowing others to build on existing research. Students completing NUTR 230 FYRE will be required to apply various OS practices throughout the project which are described in more detail later in this document. Training will be provided.

An understanding of research ethics is also critical when conducting any type of research involving human participants. In order to increase your understanding of research ethics, 1% of your final grade for NUTR 230 will involve completion of the TCPS 2 Tutorial Course on Research Ethics (CORE) (https://tcps2core.ca/welcome). **Your certificate of completion must be uploaded to Canvas by October 14th, 2020, at 11:59pm Saskatchewan Time to receive the 1% credit. You must also complete this tutorial to receive a grade for the entire NUTR 230 FYRE project. If this certificate is uploaded late, you will receive a grade of 0 for this 1% portion of your NUTR 230 course mark. Please do not email your certificate to the course instructor or research coach; it should be uploaded to Canvas.**

Feedback during the FYRE project will be provided through Canvas, email and/or WebEx at various checkpoints. We are also available to help you anytime throughout the course with this project – please ask us lots of questions! The *FYRE Lounge* in Canvas is a discussion board for

all things FYRE. Please feel free to ask questions here about anything related to FYRE. Your research coach is always your first point of contact for questions about FYRE.

You will also be provided with resources on writing survey questions, data analysis using Microsoft Excel, and research poster making. A poster template and sample poster are also available in Canvas.

A list of pre-assigned teams has been posted in Canvas. All team members should contribute equally to this project. It is best not to let one team member take over the project or to have one team member not contribute adequately. Both of these scenarios are problematic. You will have the opportunity at the end of the course to individually evaluate the contributions of your team members to this project, and confidentially submit your responses. A form has been posted in Canvas for providing these assessments. **This assessment form is due on December 6th, 2020 at 11:59pm Saskatchewan Time and is submitted via Canvas. If you do not submit this form on time through Canvas, you will receive a grade of 0% for this 2% portion of your grade for NUTR 230.**

There will be four checkpoints for this project which are described below. The first two checkpoints must occur in the first month of classes in order to allow your survey questions to be submitted to the University of Saskatchewan Behavioural Research Ethics Office for approval. We realize this is early, but in order for this project to be successful, this is necessary. At each Checkpoint, there will also be OS activities that need to be completed; these activities will be written in blue.

After some Checkpoints, we will have "NUTR 230 Rapid FYRE Updates." During a synchronous class session following some checkpoints, each team will have up to 90 seconds to provide the NUTR 230 class with an update on their research project. These updates will allow the class to be informed of what other teams are doing for their research projects.

Checkpoint #1: Submission of Research Question and Justification

The first step of this assignment is to develop a research question on practices, knowledge and/or attitudes related to nutrition, food and/or dietetics that can be investigated using survey questions in the University of Saskatchewan community. One example of a research question might be: What are the current practices surrounding use of vitamin and mineral supplements by the University of Saskatchewan community?

I encourage your team to choose to investigate a question of interest, that is current, to think creatively, and have fun with it. We do not want to assign research questions as we want you to think creatively, and choose something of interest to your group. You should not choose a question that has been chosen in the last couple of years. We will provide a list of previous NUTR 230 FYRE topics for your information in Canvas. We will help you to ensure that your question is appropriate and manageable for this assignment. If you have trouble developing a research question, please ask for guidance and we will be happy to assist.

Once your team has decided on a research question, please submit your question as well as up to 100 words that describe the reasons your team is interested in this question in Canvas. Your justification can be written in paragraph or point form.

Deadline for Checkpoint #1: Tuesday September 14th, 2020 at 11:59pm Saskatchewan Time

Please do not move further on the project until you have received approval from the instructor and/or research coach by email or via Canvas. We want to make sure that you are starting off on the right track.

Once you have approval, you will be required to complete the OS Component.

Open Science Checkpoint #1: The Open Science Framework (OSF)

Tasks

- All team members create an OSF account
- Designate a member of your team to:
  - Create an OSF project for your FYRE project
  - Add all members of the team to the OSF project
  - Make the project public
- Together, team members should:
  - Add a project title to the OSF
  - Write a description of your study in the "Description" field of the OSF homepage
  - Upload your graded Research Question and Justification as a Microsoft Word or PDF document (see file naming guidance) with the following information:
    - Title of project
    - All names of team members
    - Research Question
    - Justification/rationale
  - Add the citation to your OSF project in APA format to the Canvas Open Science Team Projects page
- **Deadline:** Tuesday September 21st, 2020 at 11:59pm Saskatchewan Time

Checkpoint #2: Submission of Survey Questions

Once you have received approval for your research question, your team will be asked to develop three to five survey questions to investigate your research question. You can use the following question types:

- yes/no
- true/false
- multiple choice (single answer allowed)
- multiple choice (choose all that apply)

Other question types (with the exception of open-ended questions) may also be approved. Note: questions capturing demographic information (e.g., age, sex, position at the University of Saskatchewan) will be part of the larger class survey and you do not need to write those. If there are specific demographic questions of interest to your team, please let us know.

Resources on writing quality survey questions have been posted in Canvas. It is also a good idea to have others (e.g., friends, family members) read your draft survey questions to make sure that they make sense and are clear. If you are asking questions about behaviours, please add a time component (e.g., Since September 2020…, In the last year…, In the last 3 months…, etc.) to your question to help improve the clarity.

Once your survey questions have been developed, please submit them in Canvas.

Deadline for Checkpoint #2: Tuesday September 28th, 2020 at 11:59pm Saskatchewan time

Your submission will be reviewed and we will schedule a WebEx meeting to meet with your team. All team members must be present at this meeting, and your questions will be finalized (or nearly finalized) at this meeting. We anticipate meeting with most teams the week of September 27th, 2020.

Once all of the questions are finalized for all teams, they will undergo review by the University of Saskatchewan Behavioural Research Ethics Board (REB) (https://research.usask.ca/rei/researchers/ethics/human-ethics.php) which can take a couple of weeks.

We will mount the questions for all of the teams along with demographic questions (e.g., sex, age, position at the University) into a single class survey on SurveyMonkey. Once ethical approval is obtained, the NUTR 230 class survey will be advertised to the University of Saskatchewan community (e.g., through PAWS). We anticipate that the entire class survey will be about 30-35 questions long. All participants will be required to provide informed consent prior to completing the survey.

Open Science Checkpoint #2: Survey Questions and Data Dictionary

Tasks

- Upload a copy of the survey questions (3-5 questions written by your team and the demographic questions you anticipate using = ~10 questions) you will be using in your study to your OSF project
- Create a data dictionary in Microsoft Excel (see template for guidance) that describes the survey data. The data dictionary must include the following for all ~10 survey questions:
    - The survey question variable name
    - The definition of the survey question
    - The type of question (e.g., text, number, multiple choice)
    - The values or ranges of the variable (e.g., numerical codes for multiple choice)
    - Instructions for how to interpret/understand the variable and/or data being collected
- Upload the data dictionary to your OSF project using .csv format (see file naming guidance)
- **Deadline:** Thursday October 7th, 2020 at 11:59pm Saskatchewan Time

Checkpoint #3: Submission of Draft Introduction and Methods Section of the Poster

This checkpoint will require you to submit the draft introduction and methods sections for your poster.

In the **Introduction** section of the poster, you need to include the following information:
-Background information on the topic (e.g., if you chose to research a question about breakfast, you might include background information on why breakfast is important, and how many people eat breakfast or do not eat breakfast etc.)
-Reason(s) your team decided to choose this research question that you did

Note: You must use 2-3 quality references to help develop the content of your introduction (e.g., recent peer-reviewed journal articles (within past ~10 years), Statistics Canada information, position papers, etc.) Please see the videos featuring Nutrition Librarian Kevin Read for some guidance on using the USask library; these videos are available in Canvas.

Hint: Most research poster introductions will have 3-5 quality bullet points

In the **Methods** section of the poster, you must include the following information (this information does not have to be included in the order provided here):

-University of Saskatchewan ethics approval statement: "This project has received ethics approval from the University of Saskatchewan Behavioural Research Ethics Board (BEH 291)"
-Number and type of survey questions developed to answer your research question; any strategies used to develop the survey questions (if applicable).
-Information on where the survey was mounted (i.e., SurveyMonkey)
-Information on how participants were recruited to complete the survey (including the dates when the survey was open to participants)
-Data analysis: Name of the computer program that your team is using for data analysis and the type of statistics that you are conducting

> Note: Everyone in NUTR 230 is using descriptive statistics which are summary values (e.g., mean, standard deviation, counts, percentages, etc.) used to describe your data

Hint: Most research poster methods sections will have 4-6 quality bullet points

Please submit your Checkpoint #3 either on a Microsoft PowerPoint poster template (.ppt or .pptx) or as a Microsoft Word document (.doc or .docx) in Canvas.

Deadline for Checkpoint #3: Thursday October 28th, 2020 at 11:59pm Saskatchewan time

We will provide feedback on your submission for Checkpoint #3 by email or in Canvas.

Open Science Checkpoint #3: Methodology

Tasks

- Upload your graded introduction and methods section to your OSF project as a Microsoft Word or PDF file (see file naming guidance)
- The document must include:
  - The title of your project
  - Team members' names
- **Deadline:** Friday November 5th, 2020 at 11:59pm Saskatchewan Time

Checkpoint #4: Data Analysis Progress and Draft Poster

Once the survey is closed to respondents, you will be provided with a Microsoft Excel spreadsheet with all of the raw data for your team's survey questions as well as demographic data. **All raw participant data must be stored on University of Saskatchewan (USask) OneDrive which is available to all students**. Details about how to access USask OneDrive have been provided in Canvas. Please ask your research coach for assistance if you need help using USask OneDrive.

*****No raw participant data can be stored on personal computers, Google (e.g., drive, docs, sheets etc.) or transmitted electronically (e.g., email) under any circumstances as this would be a violation of our course ethics approval. Only summary statistics (e.g., counts, percentages, means) and graphs can be stored on personal computers, Google (e.g., drive, docs, sheets), transmitted electronically (e.g., via email) and shared on posters.*****

Because many NUTR 230 students have not yet taken a statistics course, we will only require descriptive statistics (e.g., percentage of respondents, counts, mean etc.) for this assignment. These can all be determined in Microsoft Excel.

At Checkpoint #4, this will be a time for your team to share your progress on your data analysis and research poster. At this point, much of your data analysis should be complete, and you should be looking at putting together the Results and Conclusion sections of your poster.

Here is some key information to consider when putting together your **Results** section:

<u>Data Analysis Tips</u>:
-When calculating percentages of participants who responded in certain ways to the different survey questions, the denominator for each question will vary slightly.

- When calculating percentages, ensure that the denominator is the number of participants who answered the question that you are analyzing.
    - If participants did not answer the question that you are analyzing, they should not be included in your analysis for that specific question.
        - This means that the denominators will vary by question.
- If you are analyzing data using choose all that apply questions, the denominator is the number of participants who provided some type of answer to the overall question (i.e., checked off at least one of the options). The numerator is the number of participants who checked off the specific response option that you are analyzing.

<u>Building the Results Section in your Poster:</u>
-The first bullet point in your results section of your poster should be the data cleaning procedure. You need to provide information to readers of your poster about how many participants entered the survey (e.g., n=300), how many participants provided consent (e.g., n=298), and how many answered one or more of your team's survey questions (e.g., n=250).

-After the bullet point on the data cleaning procedure, your team should have a table that includes participant demographic information (e.g., age, sex, position at the University of Saskatchewan, etc.) This will be Table 1 for your poster and provides information to the reader about who completed your survey questions. Your demographic table should only include information on participants who answered one or more of your team's survey questions.

Here is an example of what Table 1 might look like:

Table 1: Participant Demographics

	n (%)*
**Sex**	
Male	180 (45.6)
Female	210 (53.2)
Prefer not to answer	5 (1.3)
**Position at USask**	
Undergraduate Student	300 (75.9)
Graduate Student	50 (12.7)
Staff	45 (11.4)
**Age (years)**	
≤19	200 (50.6)
20-24	100 (25.3)
≥25	95 (24.1)

*n=number of respondents; %=percentage of respondents

-Your team should then present information about how all participants answered your different survey questions. You can choose to present this information in bullet points, tables, and/or graphs (which are labeled as figures). As you move through your analysis, it will become clearer how this will look.

-Your team should also present 2-3 comparisons of how different types of participants answered one or more of your survey questions (e.g., differences between males and females for question 1, differences between students and staff for question 2, differences in undergraduate vs. graduate students for question 3 etc.) When you begin exploring your data, you will determine what types of analyses would be interesting to present.

-The word "significant" should not be used in NUTR 230 FYRE posters – this word can imply statistical significance which you did not assess. Think instead about using words like "higher" or "lower."

-Please remember to include both a label for the x and y axis in your graphs. Each graph should also have a title which could include for example: **Figure 1**: Vitamin supplement use by age.

-Your results section should include a combination of tables, text (bullet points), and graphs. It should not be only text.

-We generally recommend that when reporting numbers in tables or text that one decimal place should be used.

Here is some key information to consider when putting together your **Conclusion** section:
-There should be ~1-2 bullet points that summarize the key findings of your research
-There should be ~1-2 bullet points that compare your results to at least two other related peer reviewed research studies in a similar area
-There should be ~1-2 bullet points about what the findings mean and/or how they could be used by others
-There should be ~1 bullet point on the limitations of your research
-There should be ~1 bullet point on the next steps for this research (e.g., what would you do next time you do research on this topic).

<u>Deadline for Checkpoint #4:</u> Tuesday November 16th, 2020 at 11:59pm Saskatchewan Time

At this checkpoint, you do not need to email any files to us or upload anything to Canvas, but you should have files available for us to view in your team's USask OneDrive folder (e.g., Microsoft Excel files, draft poster). We will schedule a WebEx meeting with your team for the week of November 15, 2020 to discuss progress and answer any questions you may have. All team members must be present at this meeting. At this point, we realize each team will be at different points in their project, but we expect you to have done a substantial amount of your data analysis and be well on your way with your poster.

Open Science Checkpoint #4: Data Analysis Plan

Tasks

- Create a data analysis plan (see template for guidance) for your project that includes the following:
  - The title of the project
  - Team members' names
  - A general summary of what the data analysis plan is
  - A description of every data analysis procedure you complete, including:
    - The type of analysis performed (e.g., average, mean, count)
    - The variables you analyzed and the value(s) you explored (e.g., gender = female, coffee consumption = 5 times a day)
    - Any final figures or summary results from the analysis
  - Links to your survey and data dictionary files on your OSF project so that others can better understand your analysis
- Upload your completed data analysis plan as a Microsoft Word or PDF file to your OSF project (see file naming guidance)
- **Deadline:** Thursday November 25th, 2020 at 11:59pm Saskatchewan Time

Submission of Final Poster for Evaluation

Sections that need to be included on the final poster:

1) Title
2) Author names and affiliation
3) Introduction
4) Research Question (should be stated as a question in the poster)
5) Methods
6) Results
7) Conclusion
8) References (APA format for the reference list only. When you are citing references in the text of your poster such as the introduction or discussion, you can use numbering (bracketed e.g., (1) [1] or superscript[1]) to save space)
9) A separate reference to your OSF project using APA style, with a description of what is included in your OSF project.
10) Acknowledgements (Optional)

More information on poster content will be provided in class and discussed at individual team meetings. When creating your poster, a four column poster should be used; please see the example provided in Canvas. The FYRE logo is also posted in Canvas and can also be included near the title of the poster, if desired. Please note, research posters are a single PowerPoint slide, not two or more slides.

Please submit your posters both in .ppt or .pptx AND .pdf formats on Canvas.

Deadline for Final Poster: Thursday November 25th, 2020 at 11:59pm Saskatchewan Time [The poster submitted on this day will be the one that is graded].

At this point, we may send back your poster with minor grammar, spelling, and formatting edits for correction prior to the virtual NUTR 230 poster showcase. These edits will require a quick turnaround time.

Open Science Checkpoint #5: Final Poster

Tasks

- Include the citation for your OSF project in your final poster, indicating what is available within your OSF project
- Upload your final poster to your OSF project in PDF format (see file naming guidance)
- **Deadline:** Tuesday November 30th, 2020 at 11:59pm Saskatchewan Time

Poster Showcase

The NUTR 230 FYRE poster showcase will take place virtually during the last week of classes. More details will be provided at a later date.

FYRE GRADING COMPONENTS:

Your grade for the FYRE component of NUTR 230 will consist of the following components:

TCPS 2 Tutorial Course on Research Ethics (CORE): 1% (individual mark for on time submission of certificate of completion in Canvas)
Satisfactory Open Science Checkpoint #1 Completion: 1%*
Satisfactory Open Science Checkpoint #2 Completion: 1%*
Satisfactory Open Science Checkpoint #3 Completion: 1%*
Satisfactory Open Science Checkpoint #4 Completion: 1%*
Satisfactory Open Science Checkpoint #5 Completion: 0.5%*
Research Poster and Poster Presentation: 22.5% (team mark) (rubric will be posted later in the term). On this rubric, there will be marks for on time completion for the different FYRE checkpoints (these do not include the Open Science checkpoints). If checkpoints are not completed on time, you will lose marks on your final poster.
Summary of Your Peer's Assessment of Your Contributions: 2% (individual mark)
Total: 30% of Final Grade for NUTR 230
*: OS Checkpoint grades will either be 0 or 1. In order to receive a grade of 1, the submission must be of adequate quality (which includes completion of all components), and completed on time.

Contact Information

General questions regarding FYRE

Amanda Vanzan (Research Coach) (Your first point of contact for FYRE)
agv665@usask.ca

Jessica Lieffers
jessica.lieffers@usask.ca

Questions related to Open Science activities

Kevin Read
kevin.read@usask.ca
