# Supplementary material for: Integrating open science education into an undergraduate health professional research program
Source: J Med Libr Assoc. 2022 Oct 1;110(4):429–37. doi: 10.5195/jmla.2022.1457 (PMC10124608; doi:10.5195/jmla.2022.1457)
Supplement: Supplementary file 4 — Appendix D: Data Dictionary Template [file jmla-110-4-429-s04.pdf]

Appendix D: Data Dictionary Template

Variable Name	Variable Definition	Variable Type
age_years	The age of participants in years	text
Do you take iron supplements?	Assessment of participant's iron intake	multiple choice
What are your three favourite foods?	Participant's top three selections of their favourite foods	checkbox

Variable Values	Variable Instructions (if necessary)
N/A	Must enter a number value for age
0 - No; 1 - Yes; 2 - Don't Know	N/A
1 - Chocolate; 2 - Pizza; 3 - Steak Dinner; 4 - Sour Patch Kids; 5 - other	Other is a free text field and can be entered in manually by participants
