# Supplementary material for: Integrating open science education into an undergraduate health professional research program
Source: J Med Libr Assoc. 2022 Oct 1;110(4):429–37. doi: 10.5195/jmla.2022.1457 (PMC10124608; doi:10.5195/jmla.2022.1457)
Supplement: Supplementary file 5 — Appendix E: Reflection Assignment [file jmla-110-4-429-s05.pdf]

Appendix E: Reflection Assignment

NUTR 230
Professional Practice I
Open Science Reflection Assignment
Value: 5% of final grade
Due Date: December 7, 2020

Assignment Description:

Reflecting back on your experience with Open Science (viewing the open science lecture, uploading your NUTR 230 FYRE research outputs to the Open Science Framework, and thinking about open science as a part of the research process), please write a 250-1000 word personal reflection that addresses the following points:

a) What is your opinion of practicing open science in research?

b) What do you see as the positive aspects of open science?

c) What do you see as the negative aspects of open science?

d) Do you think you will practice open science in future research projects? Why or why not?

e) What impact (if any) do you think open science can have on research more broadly?

Marking Criteria:

Reflections will be graded based on the following criteria:

- Inclusion of all required components
- Breadth and depth of response
- Organization and clarity
- Grammar, spelling and formatting

All reflections should be double spaced, size 12 font (Calibri, Arial, or Times New Roman), 1" margins and be submitted in one of the following file formats: .pdf, .doc, or .docx.
