# Supplementary material for: Integrating open science education into an undergraduate health professional research program
Source: J Med Libr Assoc. 2022 Oct 1;110(4):429–37. doi: 10.5195/jmla.2022.1457 (PMC10124608; doi:10.5195/jmla.2022.1457)
Supplement: Supplementary file 6 — Appendix F: Aggregate Data and Codebook [file jmla-110-4-429-s06.pdf]

Appendix F: Aggregate Data and Codebook

What do you see as the positive aspects of open science?	# of students (n=21)
Trust in research/researcher	1
Preservation	2
Credit for non-traditional products of research	3
Reproducibility	4
Public good	5
Discovery	8
Receiving feedback	8
Collaboration	9
Efficiency	11
Accessibility	11
Accountability	12
Transparency	14

What do you see as the negative aspects of open science?	# of students (n=21)
Confidentiality	2
Exposure to criticism	2
Financial implications for researchers	2
Closed research culture	3
Trust in research/researcher	3
Learning curve	3
Lack of peer review	4
Lack of uptake	4
Not receiving credit	5
Misinterpretation of research	6
Being scooped	7
Time	9

**Will you consider practicing open science in the future?**	**# of students (n=21)**
Yes	19
Undecided	2
No	2
*two students provided a yes and no answer	

**Why have you decided to practice open science in the future?**	**# of students (n=21)**
Change research culture/practice	2
Transparency	2
Reproducibility	3
Open Science Framework	3
Collaboration	3
Efficiency	3
Accountability	4
Increase public access	6
Receiving feedback	7
Personal belief	8

**Why are you undecided about practicing open science in the future?**	**# of students (n=21)**
Not interested in research	1
Fine with current rules	1
Not common practice at this time	1

**Why will you not practice open science in the future?**	**# of students (n=21)**
Time	2

Pointless for school projects	2

**What impact do you think open science can have on research, broadly speaking?**	**# of students (n=21)**
Combat misinformation	1
Discovery	1
Efficiency	2
Accountability	3
Research integrity	4
Reproducibility	4
Increase public access	6
Collaboration	10
Change research culture/practice	12

Code	Response Context(s)
Accessibility	A
Accountability	A, C, D
Being scooped	B
Change research culture/practice	C, D
Closed research culture	B
Collaboration	A, C, D
Combat misinformation	D
Confidentiality	B
Credit for non-traditional products of research	A
Discovery	A, D

Efficiency	A, D
Exposure to criticism	B
Financial implications for researchers	B
Increase public access	C, D
Lack of peer review	B
Lack of uptake	B
Learning curve	B
Misinterpretation of research	B
Not receiving credit	B
Open Science Framework	C
Personal belief	C
Preservation	A

Public good	A
Receiving feedback	A, C
Reproducibility	A, C, D
Research integrity	D
Time	B
Transparency	A, C
Trust in research/researcher	A, B

Definition

When a student refers to open science as a way to provide access to research or information.
When a student refers to open science as a way to hold researchers, or themselves accountable for the work that they do.

When a student expresses concern that open science could result in their ideas or research being scooped.

When a student indicates that open science could be responsible for changing research culture or researcher practices.

When a student refers to a closed model of research, meaning the opposite of open science.

When a student refers to open science being a way to increase or foster collaboration.

When a student refers to open science as a way to combat misinformation, specifically in response news.
When a student expresses concern about open science creating risks with respect to the confidentiality of research participants.

When a student refers to open science as a way to receive recognition or credit for research products other than a publication.
When a student discusses open science as a way to improve the discovery of research products, as well as the ability for open science to increase the likelihood of new research discoveries because of access to information.

When a student refers to open science improving the efficiency of their, or others work.

When a student expresses concern that making their research open may expose them to criticism they haven't encountered before.

When a student mentions that there is a financial burden to making research open across the lifecycle.

When a student indicates that open science can increase access to research -- specifically the general public.

When a student expresses concern that open science can result in research being available but has not yet been peer reviewed.

When a student notes that open science is not yet common practice, and therefore it makes them hesitant to practice themselves.

When a student mentions that practicing open science includes a learning curve to become comfortable. This can be in the context of preparing research products to be shared and using resources.

When a student expresses concern that open science may result in others misinterpreting research either intentionally or unintentionally.

When a student indicates that receiving credit for sharing research products other than a publication is not common practice.

When a student mentions the Open Science Framework resource in either a positive or negative way.

When a student indicates they have made a decision related to open science based on their own personal beliefs or feelings about the topic.

When a student highlights that open science allows for their information to be available online for long periods, or mentions a form of preservation such as backups, long term storage, or availability in the long term.

When a student mentions that open science is a public good that would benefit society or the general population.

When a student mentions that open science provides an opportunity to receive feedback or review of their research at any point in the process.

When a student mentions that open science can lead to more reproducible research.

When a student mentions that open science can improve the integrity of research. Reasons provided may include trust in research, quality of research, and availability of research.

When a student indicates a barrier to open science is the amount of time required to practice it.

When a student mentions that open science has an effect on the transparency of research, specifically providing a clear view of how research is completed at every stage of the research process.

When a student mentions, either positively or negatively, that open science has an impact on the level of trust they have in research broadly, or individual researchers.

Example	Response Context Letter
"As aforementioned, a positive aspect of open science would include greater accessibility to knowledge . I believe that open science attracts people who desire to share their (at least preliminary) findings so that others may contribute and/or build from the research – and in turn publish their work as open access, thus continuing the cycle."	A
"the most prominent benefit of open science is that it keeps scientists accountable in regards to the accuracy, integrity, and execution of their research"	B
"Some of the negative aspects of open science can be if research is published prematurely and without proper documentation, it can be passed off as another person's research, or they can have their ideas stolen before they're able to come to a conclusion"	C
"The effect of open science on research is immense but also imperative, in my opinion. Inclusivity, intentful work, and collaboration would flourish, de-emphasizing the culture of competition and hyper-productivity that permeates academia."	D
"I view open science as being innovative, and effectively challenges the status quo of current closed research methods."	
"Open science has the potential to harness the competitiveness of academia and enable more breakthroughs through collaboration"	
"Open science may also be a way to combat the nutrition misinformation that is evident today . This will lead to the public being able to evaluate research from the source, increase nutrition knowledge, and ultimately, change how research is undertaken."	
"At first, I am concerned with how confidentiality of data and credits of researchers can be guaranteed when a project is "opened","	
"By eliminating the value of research being solely dependent and measured by the results, researchers can feel less pressured to produce desirable results. There is value in sharing all stages of the research process, and therefore open science can highlight this.?	
"people make discoveries and share their discoveries openly so that others can build off what they discovered, continually iterating scientific knowledge."	

"Open Science was extremely easy to use and it was very efficient in terms of collaborating with my groupmates. I also appreciate how organized all my files are when I upload them to Open Science."

"Posting it publicly also opens the project up to criticisms that they may not normally face in a public forum"

"However, the financial barrier is placed on research authors and their funding. Those that have less access to funding resources are put in a disadvantage when publishing their research."

"By using open science, researchers can connect with other researchers and the public."

"Another potential negative is a potential lack of professionalism: the lack of peer review or even publishing competitiveness did nothing to affirm my contributions in the context of “professional” research and thus I had no incentive to further develop my writing or poster making skills."

"Open science is not yet widely used or known about. I think this is one of its’ major downfalls."

"Another negative aspect that I personally experienced was the learning curve required due to a lack of prior knowledge and familiarity with open science and the sharing platform"

"There is potential misuse of information to create severe problems both politically and scientifically. For example, use of microbial research results to create biological weapons."

"The negative aspects of open science could include when any part of the research process or documents are replicated without due credit."

"Uploading my work to OSF made me feel much more accountable to make sure that everything I posted was correct and kept up to date if there were any changes, which is not something I have ever felt just completing an assignment for class (including a FYRE project for a different class)."

"I would be thrilled to practice open science in the future as it is a practice with which I am philosophically aligned."

"The researchers themselves also benefit because it allows them to be organized , as well as have a backup of all their documents."

"I think one of the biggest components of open science is that it can be accessed by populations regardless of demographic factors such as socioeconomic status, job title or educational qualifications."
"In addition, we were able to get feedback on certain aspects of our project. This included both layout of our project, i.e. when we first made the open science page and folders, as well as specific documents such as the data dictionary."
"By making the entire research process public, it is easier for other researchers to reproduce the results."

"When someone is aware that people can be viewing the process of their research, they maintain a standard of practice that ensures integrity when people view their documents"
"A disadvantage of open science is that it requires extra time and effort on the researcher’s part"
"The checkpoints also helped my group to focus on the minute details, such as the data dictionary and the data analysis plan, which helped us to understand our research on a more detailed scale, which by us doing this, also helps others to interpret it in an easier manner."

"In the worst-case scenario, a researcher could put out poor quality information, and a person who is unfamiliar with open science could use it as their sole information source."

Response Context

Open Science
Positives

Open Science
Negatives

Reasons IF
Practicing OS = Y

Implication of OS
on Research
